# The Integration of the Metabolome and Transcriptome for *Dendrobium nobile* Lindl. in Response to Methyl Jasmonate

**DOI:** 10.3390/molecules28237892

**Published:** 2023-12-01

**Authors:** Daoyong Gong, Biao Li, Bin Wu, Deru Fu, Zesheng Li, Haobo Wei, Shunxing Guo, Gang Ding, Bochu Wang

**Affiliations:** 1 College of Bioengineering, Chongqing University, Chongqing 400045, China; 20181901007@cqu.edu.cn; 2 Institute of Medicinal Plant Development, Peking Union Medical College, Chinese Academy of Medical Sciences, Beijing 100193, China; bwu@implad.ac.cn (B.W.); 13391950647@163.com (H.W.); sxguo@implad.ac.cn (S.G.); gding@implad.ac.cn (G.D.); 3 Steinhardt School of Culture, Education, and Human Development, New York University, New York, NY 10003, USA; df1978@nyu.edu; 4 Dehong Tropical Agriculture Research Institute of Yunnan, Ruili 678600, China; lizesheng120@163.com; 5 School of Pharmacy, Chengdu University of Traditional Chinese Medicine, Chengdu 611137, China

**Keywords:** *Dendrobium nobile* Lindl., metabolome, transcriptome, terpenoids, dendrobine, sesquiterpene synthase

## Abstract

*Dendrobium nobile* Lindl., as an endangered medicinal plant within the genus *Dendrobium*, is widely distributed in southwestern China and has important ecological and economic value. There are a variety of metabolites with pharmacological activity in *D. nobile*. The alkaloids and polysaccharides contained within *D. nobile* are very important active components, which mainly have antiviral, anti-tumor, and immunity improvement effects. However, the changes in the compounds and functional genes of *D. nobile* induced by methyl jasmonate (MeJA) are not clearly understood. In this study, the metabolome and transcriptome of *D. nobile* were analyzed after exposure to MeJA. A total of 377 differential metabolites were obtained through data analysis, of which 15 were related to polysaccharide pathways and 35 were related to terpenoids and alkaloids pathways. Additionally, the transcriptome sequencing results identified 3256 differentially expressed genes that were discovered in 11 groups. Compared with the control group, 1346 unigenes were differentially expressed in the samples treated with MeJA for 14 days (TF14). Moreover, the expression levels of differentially expressed genes were also significant at different growth and development stages. According to GO and KEGG annotations, 189 and 99 candidate genes were identified as being involved in terpenoid biosynthesis and polysaccharide biosynthesis, respectively. In addition, the co-expression analysis indicated that 238 and 313 transcription factors (TFs) may contribute to the regulation of terpenoid and polysaccharide biosynthesis, respectively. Through a heat map analysis, fourteen terpenoid synthetase genes, twenty-three cytochrome P450 oxidase genes, eight methyltransferase genes, and six aminotransferase genes were identified that may be related to dendrobine biosynthesis. Among them, one sesquiterpene synthase gene was found to be highly expressed after the treatment with MeJA and was positively correlated with the content of dendrobine. This study provides important and valuable metabolomics and transcriptomic information for the further understanding of *D. nobile* at the metabolic and molecular levels and provides candidate genes and possible intermediate compounds for the dendrobine biosynthesis pathway, which lays a certain foundation for further research on and application of *Dendrobium*.

## 1. Introduction

*Dendrobium nobile* Lindl. (*D. nobile*) is an endangered medicinal plant of the genus *Dendrobium* that is widely distributed in southwest China and is of great biopharmaceutical and horticultural importance [1]. As one of the most popular species within the genus *Dendrobium*, *D. nobile* is a fundamental plant for the production of “Shi Hu” according to *Chinese Pharmacopoeia* (2020 edition). *D. nobile* demonstrates the activity of tonifying Yin, nourishing the stomach, benefiting essence, tonifying the kidneys, treating deficiencies of the five internal organs, and so on [2]. As an ornamental and medicinal orchid, *D. nobile* is an important raw material in the development of various medicinal formulations, such as the Mailuoning injection, *Dendrobium* Yeguang pills, and *Dendrobium* Mingmu pills. The main components found in *D. nobile* are flavonoids, alkaloids, phenols, polysaccharides, sesquiterpenoids, coumarins, and steroidal glycosides [3,4,5,6,7]. *D. nobile* alkaloids (DNLA) are one of the prime active ingredients derived from *D. nobile*; they possess diverse pharmacological effects, such as forming an anti-white internal barrier, anti-tumor activity, blood sugar lowering, blood fat lowering, and nerve protection [8]. Moreover, the polysaccharides from *D. nobile* (DNPs) show a variety of biological activities, such as anti-viral [9], anti-tumor [9], anti-oxidant [10], anti-photodamage [11], and immunomodulatory [12] effects, as well as stimulating follicular development improvements [4], and protection against gastric damage [13]. Additionally, *D. nobile* extract also demonstrates the functions of whitening, moisturizing, anti-aging, and constipation reduction [14]. Hence, *D. nobile* also has good application prospects in the cosmetics industry.

Alkaloids were the earliest compounds isolated from *Dendrobium* medicinal materials. In 1932, the Japanese scholar Suzuki first isolated an alkaloid from *D. nobile*, which was named dendrobine. Subsequently, chemical synthesis studies on dendrobine were carried out by other researchers [15]. Nowadays, Dendrobine is a characteristic index component that is used for evaluating the quality of *D. nobile*. Dendrobine is a sesquiterpenoid alkaloid, and its unique picrotoxane-type structure has great functional diversity in other plants [8,16]. So far, the downstream part of the dendrobine biosynthetic pathway remains unclear. Only the upstream pathway involving mevalonate (MVA) has been elucidated [17]. Some progress has been made in the extraction, separation, structure identification, and activity evaluation of *D. nobile* polysaccharides, but the synthesis pathway, molecular mechanism, and the factors influencing polysaccharides have not yet been clarified. Methyl jasmonate (MeJA) is an organic compound that exists widely in plants. Exogenous use of 100 μM MeJA can stimulate the expression of plant defense genes, induce phytochemistry defenses, and produce similar effects to mechanical damage and insect feeding. Seven *bZIP* genes have been shown to be significantly upregulated, suggesting that these genes increase the stress resistance of Moso bamboo [18]. However, the effect of MeJA treatment on various components and contents of *D. nobile* has not been fully studied. Therefore, it is very necessary to investigate the effects of MeJA on the metabolites of *D. nobile*.

Transcriptome sequencing (RNA-Seq) technology has an overwhelmingly important role in discovering and identifying the genes involved in metabolite biosynthesis and has led to many genes involved in the biosynthesis of pharmacologically active components, such as terpenoids and alkaloids, being discovered and identified in plants [19]. For example, terpenoid-related genes from *Ipomoea batatas* [20], *Ginkgo biloba* [21], and *Scutellaria barbata* [22] have been discovered and identified. Alkaloid-related genes have been found in *Catharanthus roseus* [23], *Dendrobium officinale* [24], *Zanthoxylum armatum* [25], and so on. Combined analysis of metabolomics and transcriptomics can more comprehensively and systematically explore the regulatory mechanisms used in the biosynthesis of secondary metabolites [26]. Although some terpenoid- and alkaloid-related genes have been found in *D. nobile*, whether these genes are involved in its complete metabolic pathway is rarely reported upon. Although there have been many studies on the transcriptome of *D. nobile*, the determination and regulation mechanisms underlying the biosynthesis of the secondary metabolites induced by MeJA have not been reported [2]. Therefore, in order to explore the metabolic pathways involved in these genes, the transcriptome and metabolome of *D. nobile* were fully utilized to analyze various metabolites. The transcriptomic and metabolomics analyses showed that exogenous MeJA regulated galanthamine biosynthesis and increased jasmonate (JA) synthesis and JA signaling pathway gene expression; moreover, a significant accumulation of *Amaryllidaceae* alkaloids was found in *Lycoris longituba* seedlings [27]. Furthermore, the molecular regulatory mechanism of fatty acid biosynthesis in *Styrax tonkinensis* seeds in response to MeJA treatment was revealed by metabolomics and transcriptomics analyses [28], as was the accumulation pattern of genes involved in the synthesis of bioactive compounds (physalins, flavonoids, and chlorogenic acid) in response to MeJA in *Physalis angulata* [29]. In this study, a combined analysis of the metabolome, the transcriptome, the gene expression pattern, and metabolite differences in *D. nobile* at different stages of MeJA treatment were studied to reveal the genetic correlations among the metabolites. The results of this study will broaden the plant omics database and provide a reference for the analysis of the biosynthetic pathways of bioactive components from *D. nobile*.

## 2. Results

### 2.1. Analysis of the Dendrobine Content Following MeJA Treatment

**Figure 1:**
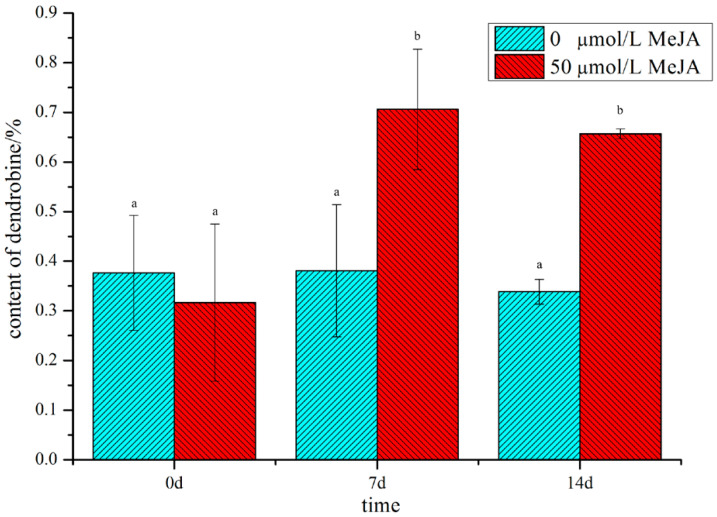
The content of dendrobine in *D. nobile* in MeJA-treated and non-treated groups. Cyan indicates the non-treated group, red indicates the MeJA-treated group, and the small letters indicate significant differences(*p*-value ≤ 0.05).

In our preliminary study with different concentrations of MeJA, we found that treatment with 50 μM MeJA significantly increased the content of dendrobine (Figure S1). The content of dendrobine reached its peak at 7 d and fell slightly at 14 d; however, the content of dendrobine in the treated group was significantly higher than that in the non-treated group (Figure 1), suggesting that MeJA could play a vital role in the accumulation of dendrobine. Therefore, in order to uncover the regulatory mechanism of *D. nobile* in response to MeJA and to explore the biosynthetic pathway of dendrobine, metabolome and transcriptome analyses were performed on *D. nobile*.

### 2.2. Metabonomic Profiling

In this study, the total ion current (TIC) of all the samples showed high overlap and stability, with a consistent retention time and peak intensity (Figure S2). Eighteen samples were selected and divided into six groups for a widely targeted metabolomic analysis with three biological replicates in each group. A total of 615 metabolites were screened and identified using the ultrahigh-performance liquid chromatography–tandem mass spectrometry (UPLC–MS/MS) platform and the MWDB (Metware database) to conduct a metabolic analysis of *D. nobile*. These metabolites were divided into 16 categories, with the order from highest to lowest being flavonoids, phenolic acids, lipids, amino acids and their derivatives, alkaloids, nucleotides and their derivatives, sugars and alcohols, organic acids, lignans, quinones, terpenes, vitamin, stilbene, tannins, xanthone, and others. The secondary metabolites accounted for a large proportion of the detected metabolites, suggesting that *D. nobile* has strong secondary metabolic activity (Table 1). According to principal component analysis (PCA), there were differences in the metabolites found in *D. nobile* after different treatment times, since these samples could be divided into different clusters (Figure S3A). As shown in Figure S3A, PC1, PC2, and PC3 could explain 20.14%, 13.61%, and 10.12% of the total variance in the samples, respectively, and the cumulative interpretation rate reached 43.87%. In addition, the heat map classification results showed that the changes in the metabolite contents in CK (samples on day 0 for the control group), DC7 (samples on day 7 for the control group), DC14 (samples on day 14 for the control group), DF (samples on day 0 for the MeJA treatment group), DF7 (samples on day 7 for the MeJA treatment group), and DF14 (samples on day 14 for the MeJA treatment group) were different and could be divided into 12 categories that were consistent with the above results (Figure S3B).

### 2.3. Identification of Differentially Accumulated Metabolites (DAMs)

Owing to the “high-dimensional and massive data” retrieved from the metabolomic analysis, it is necessary to combine univariate and multivariate statistical analyses. An analysis was conducted from multiple perspectives based on the characteristics of the data, and ultimately the differential metabolites were mined accurately and efficiently. Based on the analysis results for orthogonal partial least squares discriminant analysis (OPLS-DA), the variable importance in projection (VIP) of the OPLS-DA model obtained through a multivariate analysis could preliminarily screen for differences in metabolites between different species or tissues. Meanwhile, combining the *p*-value and/or fold changes from the univariate analysis could further distinguish differential metabolites. The DAMs in each group were determined through the above statistical analysis with threshold values of VIP ≥ 1 and fold changes of ≥2 or ≤0.5. The results of 200 random permutation and combination experiments and OPLS-DA are shown in Figure S4. The above results indicated that the model is reliable and stable and that these analysis methods could be used to screen for differential metabolites. After a pairwise comparison and analysis, a total of 377 DAMs were selected, and the DAMs in each comparison group are shown in Table 2. Among them, dendrobine (pmp001125), eucommioside (pmn001381), candicine (Hmcp009963), N-acetyl-5-hydroxytryptamine (mws0677), trigonelline (pme2268), and p-coumaroyltyramine (pmp001252) showed significant accumulation in the terpenoid- and alkaloid-related metabolic pathways. In addition, significant accumulations were also observed for D-arabitol (mws0437), L-arabito (mws0438), D-threonic acid (mws0889), melibiose (mws1333), D-glucurono-6,3-lactone (mws4175), dulcitol (pme2237), and D-glucoronic acid (pme3705), which are involved in polysaccharide metabolic pathways.

### 2.4. DAMs Identification and Enrichment Analyses

**Figure 2:**
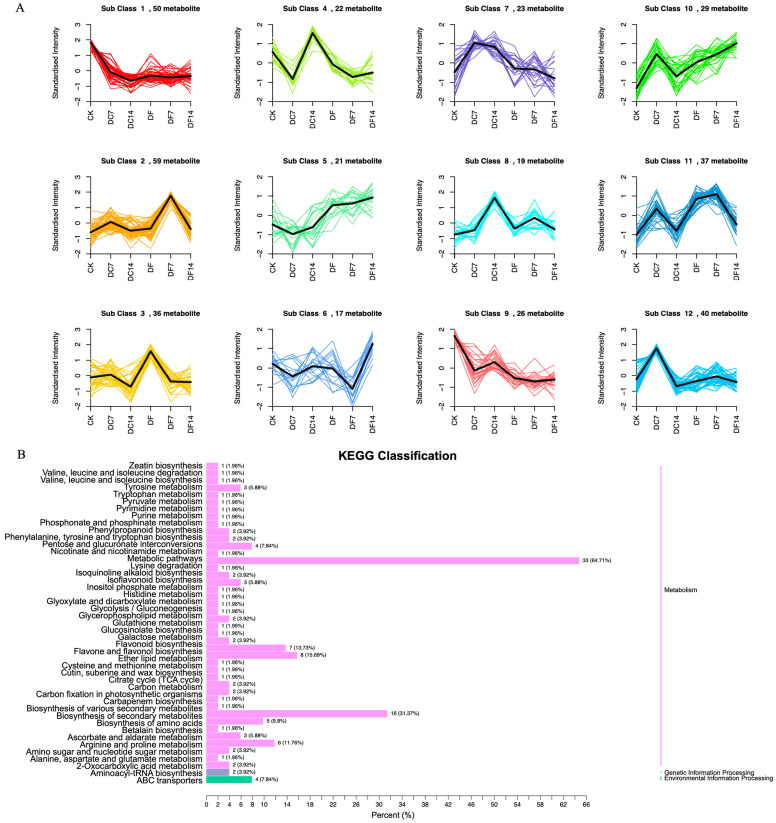
(**A**) K-means analysis of differentially accumulated metabolites (DAMs). The horizontal coordinate represents the sample name, the vertical coordinate represents the content of standardized relative metabolites, and the subclass represents the metabolites class number with the same trend. (**B**) KEGG analysis of the CK and DF14 libraries. The vertical axis shows the name of the KEGG metabolic pathway, whereas the horizontal axis shows the number of metabolites annotated to the pathway and its ratio to the total number of annotated metabolites.

The 377 DAMs could be divided into 12 clusters through a K-means analysis (Figure 2A). Among them, 59 (subclass 2), 36 (subclass 3), and 17 (subclass 6) metabolites were significantly increased in DF7, DF, and DF14, respectively. Subclass 2 mainly included flavonoids, phenolic acids, lipids, alkaloids, amino acids and their derivatives, organic acids, and quinones. Subclass 3 primarily consisted of flavonoids, phenolic acids, amino acids and their derivatives, nucleotides and their derivatives, alkaloids, and organic acids. In order to identify the functional classification of the DAMs obtained in each comparative group, the metabolites were subjected to KEGG enrichment analysis. The KEGG results indicated that each comparative group of DAMs was enriched in semblable pathways, including the biosynthetic pathways of flavones, flavonoids and flavonols, the secondary metabolites biosynthesis pathways, and the amino acid biosynthesis pathways. Compared with the CK group, DF14 exhibited a significant accumulation of 52 metabolites, which included sixteen flavonoids, eight phenolic acids, six organic acids, four alkaloids, and four lipids. The KEGG enrichment analysis results indicated that the significant DAMs between CK and DF14 were mainly concentrated in the “metabolic pathway” and “secondary metabolite biosynthesis” pathways, which accounted for 64.71% and 31.37% of the total, respectively (Figure 2B).

### 2.5. RNA Sequencing, Assembly, and Functional Annotation

A total of 132.69 Gb of clean reads were obtained after processing. The clean reads obtained from each sample reached 6.5 Gb in size, with a mean GC content of 45.59% and a Q30 base percentage of 93% or above. Subsequently, all clean reads were matched and analyzed with the *Dendrobium officinale* genome database (reference genome). Based on the comparison results, variable shear prediction, functional annotation and enrichment, gene structure optimization, and the discovery of new genes could be carried out. Ultimately, most sequences from CK (72.86–75.07%), TC7 (71.41–76.28%), TC14 (72.19–75.64%), TF (71.85–74.83%), TF7 (74.05–75.94%), and TF14 (74.17–75.11%) were matched to the *D. officinale* reference genome. At different points in time, the numbers of reads that the assembly data mapped to the “+” and “−” chains were almost equal. According to the BLASTx analysis, 16,593, 17,669, 22,782, 22,566, 55,370, 16,803, and 22,517 assembled unigenes were annotated in the GO, KEGG, KOG, NR, Pfam, Swissprot, and Trembl databases, respectively.

**Figure 3:**
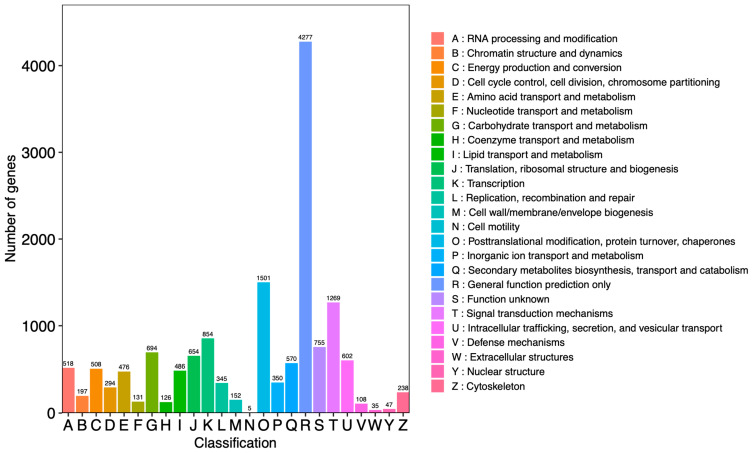
Classification of unigenes from *Dendrobium nobile* Lindl. The horizontal axis represents the functional classification ID (code) of KOG, and the vertical axis represents the number of differential genes included. Different classifications are represented by different colors. The legend is a code with a functional description.

For the evaluation of protein functions, it was generally possible to search and compare assembled unigenes from the KOG protein database to classify and identify possible functions. The results showed that a total of 15,192 unigenes were annotated and divided into 25 functional categories (Figure 3). Among these categories, the number of “general function prediction only” (R: 4277 unigenes) was the highest and accounted for 28.15% of the total.

In addition, “post-translational modification, protein turnover, chaperones” (O: 1501 unigenes) and the “signal transduction mechanisms” (T: 1269 unigenes) accounted for 9.88% and 8.35% of the total, respectively. The category “extracellular structures” (W: 35 unigenes) accounted for 0.23% of the total, which was the lowest percentage.

### 2.6. Identification and Functional Enrichment Analyses of DEGs

**Figure 4:**
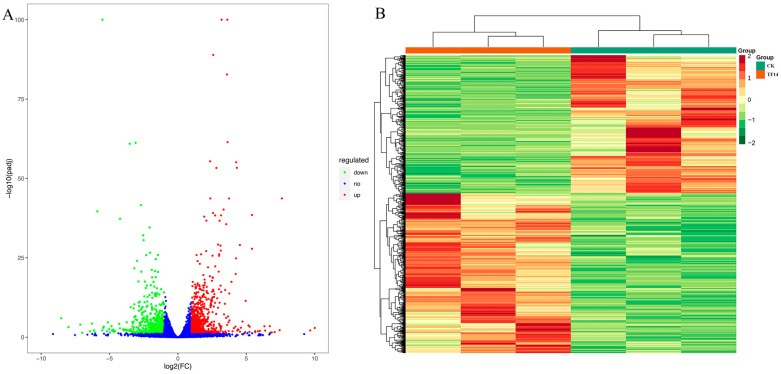
Analysis of differentially expressed genes between the CK and TF14 libraries. (**A**) Volcanic map of differentially expressed genes. The red dots, green dots, and blue dots represent significant upregulation, downregulation, and no significant difference in genes levels, respectively. (**B**) Hierarchical clustering heat map of differentially expressed genes. The *x*-axis represents the results of the differential genes and hierarchical clustering, and the *y*-axis represents the sample name and hierarchical clustering results. Red and green represent high and low gene expression, respectively.

For the biological replication samples, DESeq2 was used for the differential expression gene (DEG) analysis between sample groups. After the differential expression analysis, it was necessary to perform multiple hypothesis test corrections on the hypothesis test probability (*p*-value) to obtain the false discovery rate (FDR) using the Benjamini–Hochberg method. The fragments per kilobase of the exon model per million mapped fragment (FPKM) values were calculated for each unigene by setting | log_2_ fold change | ≥ 1 and FDR < 0.05 as the DEGs’ cut-off values. The results showed that a total of 3257 significant DEGs were screened out among the 11 comparison groups (Table 2). Among them, compared with the CK group, the largest number of significant DEGs (624 downregulated and 722 upregulated) was found in the TF14 library (Figure 4A). The hierarchical clustering of the DEGs of the CK and TF14 comparison groups is shown in Figure 4B. From the clustering heat map, it can be seen that the gene expression patterns in the TF14 and CK groups had significant differences. Similar results were observed for several comparisons, including TC7 vs. TC14, TF7 vs. TF14, and CK vs. TC14, where there were 1362, 889, and 853 DEGs, respectively. This may be due to the sampling time, hormonal induction, and individual differences in the samples. In order to study the expression patterns of genes after different lengths of MeJA treatment, a cluster analysis was used to cluster genes with the same or similar expression patterns into classes to identify the unknown functions of known genes or the functions of unknown genes. Firstly, the FPKM values of the genes were centralized and standardized, then a K-means cluster analysis was performed to determine the expression patterns of differential genes for different treatment times, and they were plotted (Figure S5).

**Figure 5:**
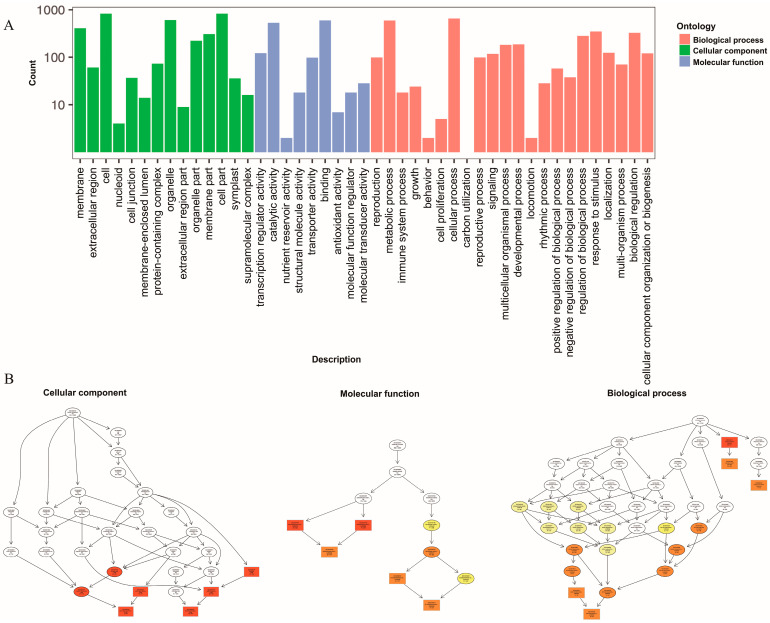
Functional GO and KEGG pathway classification of differentially expressed genes. (**A**) The horizontal axis represents the enriched GO term, and the vertical axis represents the number of differentially expressed genes. (**B**) The three main categories of the directed acyclic graphs are displayed in the thumbnail view. The node colors are based on the q-values. The GO terms appear at the horizontal node locations; the darker the color, the higher the confidence level.

**Figure 6:**
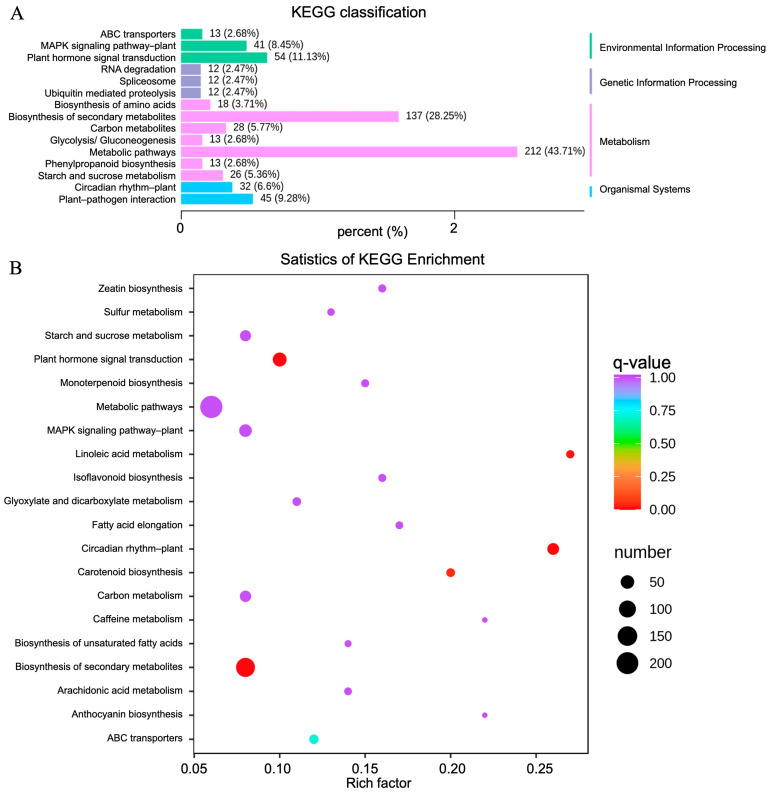
KEGG classification and enrichment analysis for the CK and TF14 libraries. (**A**) The horizontal and vertical axes represent the ratio of genes annotated to this pathway to the total number of annotated genes and the name of the KEGG pathway, respectively. The label on the right side of the figure represents the classification to which the KEGG term belongs. (**B**) The vertical and horizontal axes represent the KEGG pathway and the enrichment factor, respectively, where the larger the enrichment factor, the higher the degree of enrichment; the larger the dot, the more differentially enriched genes there are; and the redder the dot, the more significant the enrichment.

In order to better explore the biological functions of DEGs, significant DEGs in the 11 comparative groups were functionally classified and annotated through GO and KEGG enrichment analyses. For the GO analysis (Figure 5A), the significant DEGs in the “cellular component” category between the CK and TF14 libraries were mainly enriched for the “cell and cell part”, “membrane and membrane part”, and “organelle and organelle part” subcategories; the significant DEGs in the “molecular function” category between the CK and TF14 libraries were mainly enriched for the “transcription regulator activity”, “catalytic activity”, “transporter activity”, and “binding” subcategories; and the significant DEGs in the “biological process” category between the CK and TF14 libraries were mainly enriched for the “developmental process”, “metabolic process”, “cellular process”, “multicellular organismal process”, “regulation of biological process”, and “response to stimulus” subcategories. The “cellular component” category was mainly composed of upregulated DEGs, whereas the “cell wall biogenesis and hemicellulose metabolic process” subcategory was mainly composed of downregulated DEGs. Directed acyclic graphs were also used to describe similar classification results for the GO structures (Figure 5B). Three GO directed acyclic graphs were constructed, representing cellular components, biological processes, and molecular functions. In addition, 200 of the GO categories were significantly affected in the CK and TF14 libraries (Supplementary Table S1). For the KEGG functional classification, the significant DEGs between the CK and TF14 libraries were mainly enriched for the “biosynthesis of secondary metabolites”, “metabolic pathways”, and “plant hormone signal transduction” pathways, which accounted for 43.71%, 28.25%, and 11.13% of the total, respectively (Figure 6A). In order to identify the DEGs involved in the *D. nobile* metabolic pathway, we utilized analysis software from the KEGG database (v0.7.2 DTD) to identify DEG-rich pathways. The KEGG enrichment analysis showed that “metabolic pathways”, “biosynthesis of secondary metabolites”, and “plant hormone signal transduction pathways” were the most active pathways (Figure 6B); these results were similar to the KEGG functional classification.

### 2.7. Identification of Unigenes Related to the Biosynthetic Pathways of Terpenoids and Polysaccharides

In this study, 189 candidate unigenes related to the biosynthetic pathways of terpenoids were screened and identified from the *D. nobile* transcriptome. For genes related to the biosynthesis of terpenoids, 52, 39, 46, and 52 unigenes were involved in terpenoid backbone (ko00900), sesquiterpenoid and triterpenoid (ko00909), monoterpenoid (ko00902), and diterpenoid (ko00904) biosynthesis, respectively. Among them, seven unigenes were significantly upregulated in DF14; these genes were the (−)-α-terpineol synthase gene (*Loc110100458*, *Loc114579671*, *Loc110092374*, *Loc110092382*, and *Loc110097122*), the geraniol 8-hydroxylase gene (*LOC110098600*), and the squalene monooxygenase gene (*LOC110095241*). The correlation analysis (| r | ≥ 0.8) showed that a total of 238 transcription factors (TFs) were identified that may participate in the regulation of sesquiterpenoid and/or triterpenoid biosynthesis. These TFs included twenty-four MYB and MYB-related TFs, sixteen zinc finger proteins, one ZAT11, one YABBY, eight WRKY, two TINY, four TCP, one SlARF2A, one SBH1, one RINGLET, two REVEILLE, two PTI, two PRR, two protein indeterminate domains, two HK3, one PEP, one PCF, six NF-YA/B/C, three SNAT2, thirteen NAC, one MYC2, one multiprotein-bridging factor 1c, two MTERF, one MADS, one lysine-specific demethylase JMJ30, three AUX/IAA, one LOB, one LATERAL ROOT PRIMORDIUM 1, three KNAP/KAN, four JAZ, twelve HD-ZIP, six Hsf, one GT-2, one GRF6, four GRAS, one GLK1, one GHD7, five GATA, one GAG2, two FAR1, eight ERF, seven EREBP, one E2FB, two DREB2A, ten DOF, one DIVARICATA, one CPRF2, one BZR1, eight bZIP, six BTB/POZ, eight bHLH, four BEL1, five B-box, six B3, one AS2, one YABBY 2, two TCP, one RAD5A/SMARCA3, two PCF, three ORR, one NPR5, one ATXR7, and eleven other TFs (Supplementary Table S2). Of these, after stimulation with MeJA, the TFs that were increased were mainly the WRKY, NAC, MYB, HD-ZIP, ERF/EREBP, bZIP, bHLH, BTB/POZ, and DOF TF families.

In this paper, 99 candidate unigenes related to the biosynthetic pathways of polysaccharides were screened and identified from the *D. nobile* transcriptome. For genes related to the biosynthesis of polysaccharides, 56, 24, 22 and 20 unigenes participated in starch and sucrose metabolism (ko00500), amino sugar and nucleotide sugar metabolism (ko00520), fructose and mannose metabolism (ko00051), and galactose metabolism (ko00052), respectively. The correlation analysis showed that a total of 313 TFs were identified that may participate in the regulation of polysaccharide biosynthesis. These TFs included two ZHD, two ZAT11, two YABBY, fifteen WRKY, one WIP2, two TINY, six TCP, one SUVR4, two STOP, one squamosa promoter-binding-like protein 12, one SNAT2, one SHR2, one SCR, four SCL, one SBH1, one RSM2, two REVEILLE, one RAP2, one RADIALIS, one RAD5a, two PTI6, one pTAC14, three PRR, one protein indeterminate domain 2, three PCF2, one OSH1, four ORR, one NPR1, one nodulation-signaling pathway 2 protein, one ATXR7, six NF-YA/B/C, one NATA1, one NAT1, seventeen NAC, thirty-four MYB and MYB-related, one multiprotein-bridging factor 1c, three MTERF, three MADS, six AUX/IAA, one LATERAL ROOT PRIMORDIUM 1, three KNAP/T, two KAN, four JAZ, one indeterminate domain 7, six Hsf, one HMG1/2-like protein, two HLS1, three HK, twelve HD-ZIP, one HD1, one HB29, one GT-2, two GRF6, one glycine-rich protein 2, one GLK1, one GIS, one GHD7, six GATA, one GAG2, two FAR1, one ETR2, one ETHYLENE INSENSITIVE 3-like 1 protein, seventeen ERF/EREBP, one E2FB, four DREB, thirteen DOF, one DIVARICATA, one DDT, one CPRF2, six CONSTANS-LIKE, five CCCH, one lix-loop-helix protein A, eight B3, two AS2, one ARG13, one ARF6b, one APRR5, and fifteen other TFs (Supplementary Table S3). Of these, after stimulation with MeJA, the TFs that were increased were mainly the WRKY, NAC, MYB, HD-ZIP, ERF/EREBP, DOF, bZIP, BTB/POZ, bHLH, and B3 TF families.

### 2.8. qRT-PCR Validation of Differentially Expressed Genes

**Figure 7:**
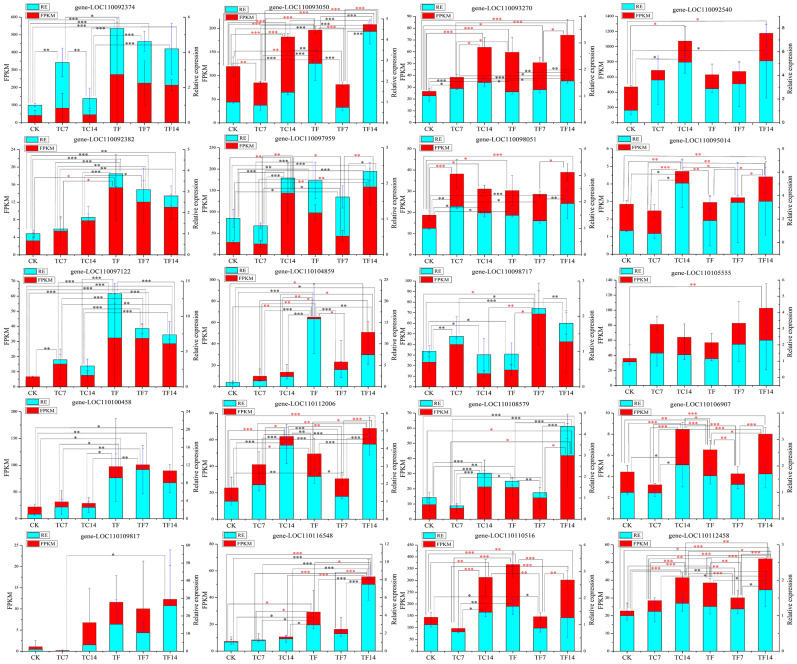
qRT-PCR analysis of differentially expressed genes in the terpenoid and alkaloid biosynthesis pathways. The *X*-axis represents the average change in the expression value, the *Y*-axis represents different expression patterns at six time points, and the FPKM values represents the average transcriptome sequencing value. The FPKM values are shown in red, and the relative expression (RE) levels are shown in cyan. A red asterisk indicates that the FPKM value is significant; a black asterisk indicates that the RE is significant; *, ** and *** represent significant *p*-values ≤ 0.05, 0.01, 0.001, respectively.

To confirm the accuracy of the transcriptome sequencing results, twenty differentially expressed genes (DEGs) involved in the terpenoid and alkaloid metabolic pathways were subjected to quantitative real-time PCR (qRT-PCR) analysis. The primers of the twenty randomly selected DEGs defined by primer premier 5.0 are listed in Supplementary Table S4. The qRT-PCR results showed that the expression levels of all these twenty DEGs were consistent with the trend for the transcriptome data (Figure 7). The correlation between the FPKM values of the different genes and the qRT-PCR data were analyzed using Pearson’s correlation coefficient (r) [30]. Among them, five DEGs reached a significant level (*p* ≤ 0.05) and the other fifteen DEGs reached an extremely significant level (*p* ≤ 0.01) (Supplementary Table S5). In order to explore the hysteresis effect of the genes and metabolites, the relative expression of the above 20 DEGs was further analyzed. We collected samples at 0 h, 0.5 h, 1 h, 2 h, 4 h, 8 h, 16 h, 24 h, 7 d, and 14 d and carried out an analysis of the relative expression levels. Some genes and metabolites were found to have a certain lag effect (Figure S6). In the relative expression analysis for the terpene synthase genes (*LOC110109817*), we found that the difference between the MeJA-treated group after 14 days of treatment (TF14) and the control group (CK) was the most significant—more than 25 times greater. However, due to the hysteresis effect, the gene *LOC110109817* was significantly overexpressed at 16 h (2399.8 times greater expression than the control group) (Figure S6). BLAST analysis using the NCBI tool revealed that this gene was (±)-germacrene D synthase (GDS). Farnesyl diphosphate (FPP) was catalyzed by GDS to produce (±)-germacrene D. Previous research has shown that germacrene D is a chiral compound that is present as two enantiomers produced by FPP via enantiomer-specific synthases [31] (Figure S7).

### 2.9. Correlation Analysis of the Metabolome and Transcriptome Data

**Figure 8:**
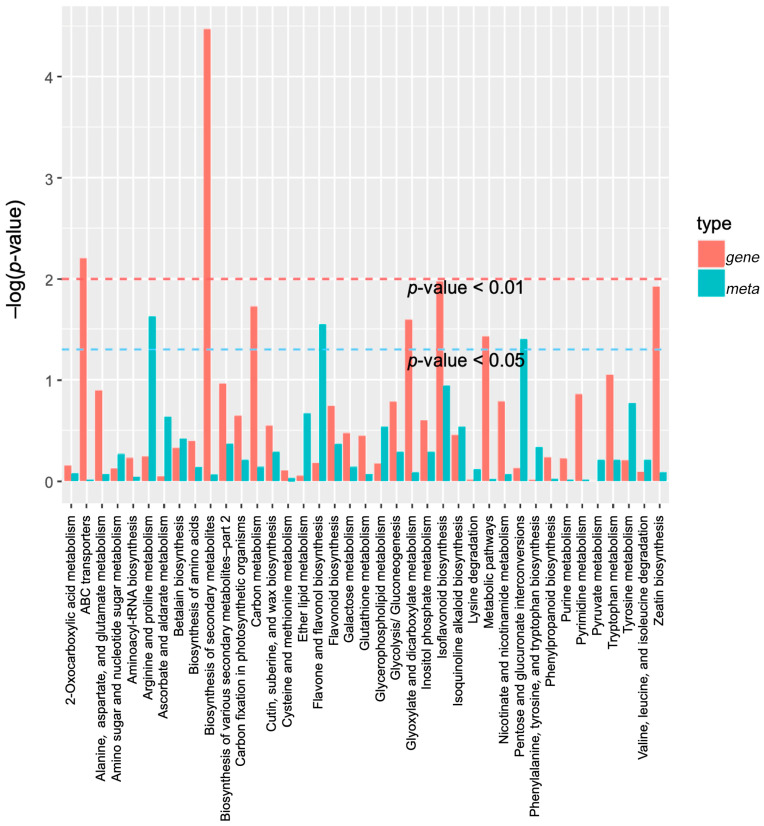
A KEGG enrichment analysis was performed for the DEGs (red column) and DAMs (green column) enriched by the same pathway. In the vertical coordinate, red and green represent the enrichment values of differential genes and differential metabolites, respectively, which are represented by the −log(*p*-value), where the higher the enrichment degree, the higher the ordinate.

**Figure 9:**
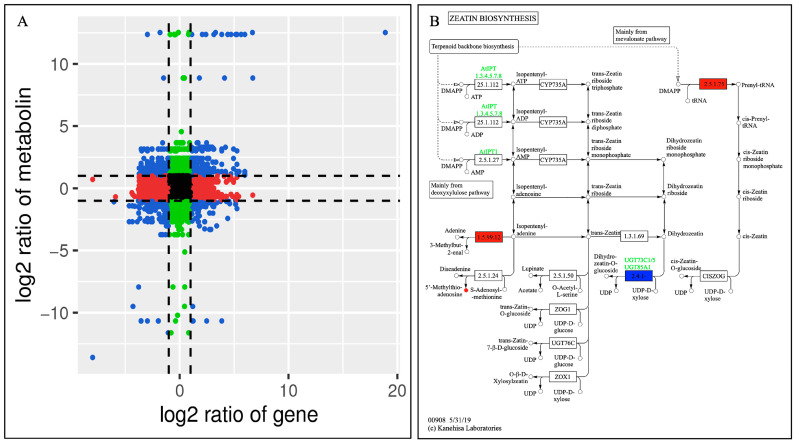
(**A**) The correlations between the DEGs and DAMs of the CK and DF14 libraries are shown in the nine-quadrant diagram. The nine quadrants are divided into one to nine quadrants from the left to right and top to bottom. Quadrant 5 represents the non-differential expression of both genes and metabolites. Quadrants 1, 2, and 4 show that the gene expression abundance was lower than the metabolite expression abundance. Quadrants 3 and 7 show that the metabolite differential expression pattern was consistent with the gene differential expression pattern. Quadrants 6, 8, and 9 indicate that the abundance of the metabolite expression was lower than that of the gene expression. Black dots indicate non-differentiated metabolites and genes, blue dots indicate both metabolites and genes with significant differences (up-regulated or down-regulated), red dots indicate genes with significant differences but metabolites with no significant differences, and green dots indicate metabolites with significant differences but genes with no significant differences. (**B**) Annotations of the differentially accumulated metabolites and differentially expressed genes in terpenoid skeleton biosynthesis. Red and green indicated that the gene or metabolite is significantly upregulated and downregulated, respectively, whereas blue indicates that the gene is upregulated and downregulated simultaneously.

The enrichment results of the differential metabolites and differential genes were analyzed using KEGG pathway analysis. Then, a histogram was drawn to show the enrichment degrees of both. Finally, by comparing the DAMs and DEGs of each group, many DAMs and DEGs were found to be enriched for the same KEGG pathway. For example, compared with CK, the pathways in the DF14 group mainly included the metabolic pathways of 2-oxocarboxylic acid, ABC transporters, carbon, tyrosin, and inositol phosphate, as well as the secondary metabolites, amino acids, flavonoid, isoflavonoid, isoquinoline alkaloid, and the flavone and flavonol biosynthesis pathways (Figure 8). A total of 33 DAMs and 212 DEGs were found to be enriched for the same metabolic pathway. A total of 16 DAMs and 137 DEGs were found to be enriched for the biosynthesis of secondary metabolites (Supplementary Table S6). A correlation analysis between the DAMs and DEGs of each group was carried out to further analyze the DAM regulation network. The correlation analysis showed that these metabolites were positively and/or negatively simultaneously regulated by multiple genes (Figure 9). Among them, a total of 492 genes were consistent with the expression pattern of the metabolites, and the changes in the metabolites may be positively or negatively regulated by these genes. The combined transcriptome and metabolome analysis showed that five DEGs were significantly correlated with four DAMs (Figure S8).

### 2.10. Dendrobine Biosynthetic Pathway Activation in Response to MeJA

**Figure 10:**
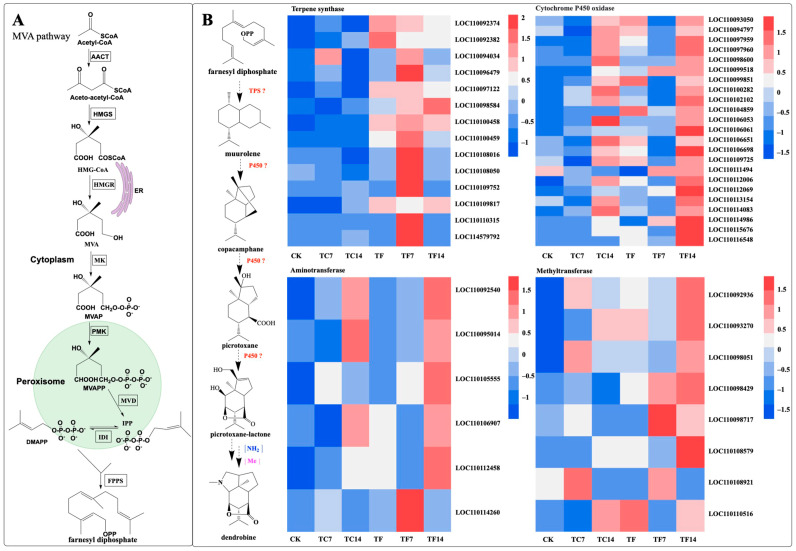
Putative biosynthetic pathways of dendrobine in *D. nobile*. A: the MVA (**A**) pathway. (**B**) the biosynthetic pathways of dendrobine and heat map of different genes. Acetoacetyl-CoA thiolase (AACT); 3-hydroxy-3-methylglutaryl- CoA synthase (HMGS); 3-hydroxy- 3-methylglutaryl-CoA reductase (HMGR); mevalonic acid (MVA); mevalonate kinase (MK); mevalonate 5-phosphate (MVAP); phosphomevalonate kinase (PMK); mevalonate diphosphate (MVAPP); mevalonate diphosphate decarboxylase (MVD); isopentenyl diphosphate (IPP); dimethylallyl diphosphate (DMAPP); isopentenyl diphosphate isomerase (IDI); farnesyl diphosphate synthase (FPPS); endoplasmic reticulum (ER); terpene synthase (TPS); cytochrome P450 oxidase (P450); NH_2_, amination reaction; and Me, methylation reaction. The expression levels of genes at the six time points are represented by heatmaps, in which the blocks shown from left to right represent CK, TC7, TC14, TF, TF7, and TF14.

Dendrobine is a terpenoid alkaloid. Terpenoid alkaloids are a group that contains sesquiterpenoid alkaloids. Studies have shown that the MVA pathway participates in the dendrobine biosynthesis pathway [32]. In order to explore whether MeJA could regulate the biosynthesis of dendrobine in *D. nobile* seedlings, we investigated the expression levels of the dendrobine-biosynthetic-pathway-related genes induced by MeJA at different time points. In the upstream part of the biosynthetic pathway of dendrobine, nine key enzyme genes (acetoacetyl-CoA thiolase, AACT; 3-hydroxy-3-methylglutaryl-CoA synthase, HMGS; 3-hydroxy-3-methylglutaryl-CoA reductase, HMGR; mevalonate kinase, MK; phosphomevalonate kinase, PMK; mevalonate diphosphate decarboxylase, MVD; geranyl diphosphate synthase, GPPS; farnesyl diphosphate synthase, FPPS; and terpene synthase, TPS21) in the transcriptome of *D. nobile* induced by mycorrhizal fungi were identified and characterized. In the downstream biosynthetic pathway of dendrobine, 144 candidate genes were identified in the transcriptome, including 19 terpene synthase genes, 66 cytochrome P450 oxidase genes, 48 methyltransferase genes, and 11 aminotransferase genes. After MeJA treatment, fourteen terpene synthase genes, twenty-three cytochrome P450 oxidase genes, eight methyltransferase genes, and six aminotransferase genes were found in the downstream pathway and may be involved in dendrobine biosynthesis (Figure 10).

### 2.11. Functional Validation of DnSQS-2

**Figure 11:**
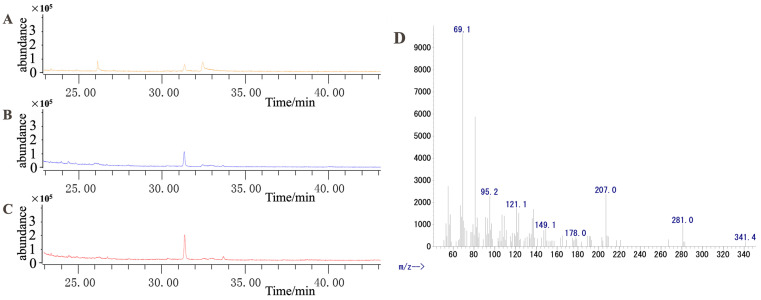
GC–MS was used to detect the catalytic product. (**A**) Product extracted from yeast engineering bacteria (WAT11). (**B**) Product extracted from yeast engineering bacteria (WAT11/pESC-TRP). (**C**) Product extracted from yeast engineering bacteria (WAT11/pESC-TRP::DnSQS-2). (**D**) Mass spectrum of the 33.622-min peak in (**C**).

Because the terpene synthase gene (LOC110109817, here named SQS-2) was highly responsive to MeJA (Figure 7), we speculate that this gene may play a vital role in dendrobine biosynthesis upon exposure to MeJA. To validate our hypothesis, this gene was cloned into yeast engineering bacteria (WAT11/pESC-TRP::DnSQS-2). The results of gene cloning and the double enzyme digestion of the recombinant plasmid are shown in Figure S9. After galactose-induced fermentation, the extracted product was measured using GC–MS (Figure 11). The results show that DnSQS-2 can catalyze the production of the moniterpenoids (C10) geraniol, cis-2,6-dimethyl-2, and 6-octadiene; the sesquiterpenes (C15) farnesol and (3S,6E)-nerolidol; the diterpenoid (C20) geranylgeraniol; the triterpenoids (C30) ambrein and squalene; and the tetriterpenoid (C40) carotene. In contrast, no product peak was detected in the yeast engineering bacteria WAT11 and WAT11 (pESC-TRP). These results indicate that DnSQS-2 may be a multifunctional enzyme.

## 3. Discussion

### 3.1. Metabolome Analysis of D. nobile

Metabolomics is a new subject that has developed rapidly after genomics and proteomics and is an important part of systems biology [33]. Metabolomics is widely used for the (qualitative and quantitative) analysis of small molecular metabolites (with a relative molecular mass of less than 1000) in organisms or cells. The main chemical constituents isolated from *D. nobile* were alkaloids, phenanthrene, bibenzyl, sesquiterpenoids, and other types [16]. The *Dendrobium nobile* alkaloid is one of the main active ingredients in *D. nobile* and has anti-cataract, anti-tumor, nerve protective, hypoglycemic, hypolipemic, and anti-oxidation pharmacological effects [34,35,36,37]. Moreover, sesquiterpenes have been reported to be present, which are represented by the special structure of the picrotoxane-type. At the same time, it was found that the sesquiterpenes of *D. nobile* have neuroprotective, immunomodulatory, and anti-tumor physiological activities [5]. As a relatively new research field in the post-genomic era, metabolomics has been effectively applied to assess the changes in metabolites between different tissues, species, or developmental stages [33,38]. In this paper, the metabolites of *D. nobile* seedlings treated with MeJA were identified and analyzed comprehensively and systematically using UPLC–MS/MS metabolomics. A total of 615 metabolites were analyzed and identified in this article, including forty-one alkaloids, nine terpenoids, and fifteen polysaccharides. These results indicate that *D. nobile* has strong alkaloid metabolic and polysaccharide activities, findings that are consistent with the transcriptomic analysis results. However, the lack of a structural analysis of the compounds in the potential pathways suggests that UPLC–MS/MS cannot easily, quickly, and systematically identify the structures of the compounds in unknown pathways without standards. As one of the four basic substances of life, polysaccharides widely exist in higher plants, animals, microorganisms, lichens, and algae [39]. Polysaccharides play anti-tumor, anti-inflammatory, anti-viral, hypoglycemic, anti-aging, anti-coagulant, immune stimulatory, and other bioactive roles [40]. Natural polysaccharides isolated from fungi and plants are used in the food and pharmaceutical industries because of their non-toxic and negligible side effects, as well as their medicinal value. The derivatives of immunologically active polysaccharides often have other activities as well [41]. Therefore, the research and development of polysaccharides has become a hot spot in recent years. The results of this paper provide reference values for the development of *D. nobile* as a raw material.

### 3.2. Transcriptome Analysis of D. nobile

The object of transcriptomics is usually the sum of all the RNA that can be transcribed by a cell in a given state and includes messenger RNA (mRNA) and non-coding RNA. This is a common experimental method for discovering new genes, quantifying gene expression, and identifying transcripts [42]. For example, many genes associated with alkaloids and terpenoids have been analyzed and identified by transcriptome sequencing in *Erythrina velutina* Willd. [43] and *Panax ginseng* [44]. Although some genes (sesquiterpene synthases and post-modification enzymes) related to terpenoid biosynthesis have been identified in *D. nobile* infected with the mycorrhizal fungus MF23 (*Mycena* sp.) [17], the genes related to alkaloid and terpenoid biosynthesis induced by MeJA have not been elucidated. In this study, on the basis of mycorrhizal fungus MF23 induction, *D. nobile* was treated with MeJA to screen genes related to the dendrobine biosynthetic pathway. A terpene synthase gene (Loc110109817) was obtained via qRT-PCR. The gene was highly expressed after MeJA treatment and reached its peak at 16 h. A sequence alignment analysis indicated that the gene was probably (±)-germacrene D synthase, ɑ-humulene synthase, or β-patchoulene synthase, and the enzyme was identified as sesquiterpene synthase [31]. FPP was catalyzed by sesquiterpene synthetase to form a sesquiterpene skeleton, e.g., (±)-germacrene D, α-humulene, and β-patchoulene. In this study, it was found that the expression level of the gene Loc110109817 (SQS2) was positively correlated with the biosynthesis of dendrobine. Therefore, we speculate that SQS2 may be a sesquiterpene synthase that is involved in the biosynthesis of dendrobine. After MeJA induces gene changes in *D. nobile* plants, SQS2 might directly or indirectly affect the biosynthesis of dendrobine. In another study, the *DnSQS-2* gene was introduced into and overexpressed in *Saccharomyces cerevisiae* and the fermentation products were analyzed. It was found that DnSQS-2 was a multifunctional synthase that can catalyze the formation of monoterpenes, sesquiterpenes, diterpenes, triterpenes, and tetriterpenes. Of these, the sesquiterpene compound farnesol may be an intermediate compound in the synthesis pathway of dendrobine. TFs usually regulate the progression of genes, thereby controlling a variety of cellular processes and cellular states [45]. It is well known that TFs usually need to cooperate with RNA polymerase Ⅱ to form a transcription initiation complex and then participate in the process of transcription initiation. Co-expression analysis indicated that 238 and 313 TFs may participate in the regulation of terpenoid and polysaccharide biosynthesis, respectively. Of these, ZAT11, NAC, MYB, ZIM, HD-ZIP, and bHLH are positively correlated with SQS2 levels, whereas B3, WRKY, CCCH, EREBP-1, BEL1, GRF6, and BTB/POZ are negatively correlated with SQS2 levels (Supplementary Table S2).

In addition, various reports have demonstrated that there may be many types of sesquiterpene synthases in the same plant species and that these enzymes compete with each other for the common precursor (FPP) to synthesize various types of sesquiterpenes when consumed [17]. In this study, we screened a sesquiterpene synthase (*TPS21*) from the transcriptome of *D. nobile* infected with the mycorrhizal fungus MF23 and found that this enzyme was negatively correlated with the biosynthesis of dendrobine. Therefore, inhibiting or reducing the expression levels of *TPS21* may lead to more FPP flowing into the dendrobine biosynthetic pathway. Similarly, increased *SQS2* expression levels may also lead to more FPP flowing to the dendrobine biosynthesis pathway. After the treatment of *D. nobile* by MeJA or the mycorrhizal fungus MF23, *SQS2* and *TPS21* may directly or indirectly affect the biosynthesis of dendrobine.

In the polysaccharide synthesis pathway, a total of 99 genes were found to be regulated by TFs after the treatment of *D. nobile* with MeJA. Compared with the *Dendrobium officinale* Kimura et Migo (*D. officinale*) polysaccharide pathway, there have been few studies on the mechanism of the *D. nobile* polysaccharide pathway. Research shows that among the 19 species of *Dendrobium*, *D. officinale* has the highest polysaccharide content, up to 22.49%, and *D. nobile* has the highest alkaloid content, up to 43.6 mg/100 g [46]. As one of the main active substances, the polysaccharides in *Dendrobium nobile* (DNP) have been shown to have anti-inflammation, anti-tumor, anti-oxidation, anti-aging, anti-diabetes, and liver protective pharmacological activities. Polysaccharides will undoubtedly become one of the best raw materials for the development of health products and have great development value. At present, some progress has been made in the extraction, separation, structure identification, and activity evaluation of DNPs; however, there are still many unknowns regarding the structure, mechanism, and efficacy evaluation of the polysaccharides. On the one hand, the extraction, purification, and structural characterization of DNPs are important prerequisites for the industrialization of DNPs. However, the extraction, separation, and purification methods for DNPs are still immature and the quality control index and standard constructions are not perfect, so there are some differences in the structures and activities of the polysaccharides obtained. On the other hand, there have been many studies on the structures of DNPs, but few of them have actually obtained the primary structure. The structure–efficacy relationship between the structural characteristics of polysaccharides and the various activities of DNPs is still in the preliminary research stage. This is the focus of future research on DNPs, and a lot of research work is required to supplement and confirm it. In addition, studies on the bioactivity of polysaccharides from *D. nobile* are still in the initial stage of exploration in cells, organs and tissues, and the molecular mechanisms of many target sites still need to be further explored. Therefore, the establishment of efficient and stable separation and purification methods, quality standards, and further studies on the structural characteristics, pharmacological activities, and molecular mechanisms of polysaccharides from *D. nobile* will be of great value for the comprehensive development and utilization of *D. nobile* resources and the extension of the industrial chain.

### 3.3. Metabolome and Transcriptome Analysis of the Metabolite Biosynthesis Pathway

The correlation between metabolites and genes was represented by a network diagram; a total of four metabolites were found to be positively correlated with related genes. These metabolites were mws4002, dopamine (alkaloids); mws2125, phosphoenolpyruvate (organic acids); pme0516, inositol (saccharides and alcohols); and mws0853, sinapyl alcohol (phenolic acid). It is well known that genes with different functions will form different metabolites and that different metabolites are related to genes with different functions. Four common KEGG pathways were found in this study. The polyphenol oxidase (PPO) genes (*LOC110105187* and *LOC110105188*) and dopamine are co-enriched in the tyrosine metabolism (ko00350) pathway, and both showed an upward trend. *PPO* encodes polyphenol oxidase, which was also associated with catechol oxidase activity (GO:0004097), the pigment biosynthetic process (GO:0046148), the chloroplast thylakoid lumen (GO:0009543), and metal ion binding (GO:0046872). It catalyzes the oxidation of o-diphenols and/or monophenols to form o-quinones while reducing oxygen to water, resulting in protein complexation and the formation of brown melanin pigments [47]. The acyl-coenzyme A oxidase 4 (ACX4) gene (*LOC110113353*) and phosphoenolpyruvate are co-enriched in the carbon metabolism (ko01200) pathway, and both showed a downward trend. *ACX4* encodes acyl-coenzyme A oxidase 4, which was also associated with glyoxysome (GO:0009514), acyl-CoA dehydrogenase activity (GO:0003995), peroxisome (GO:0005777), flavin adenine dinucleotide binding (GO:0050660), embryo development ending in seed dormancy (GO:0009793), short-chain fatty acid metabolic processes (GO:0046459), and fatty acid β-oxidation (GO:0006635). The decreased *ACX4* expression in several *Arabidopsis* backgrounds showed a differential response, indicating that the *ACX4* gene and/or protein have different functions in *Arabidopsis* accessions [48]. The galactinol synthase (*GolS*) gene (*LOC110114431*) and inositol are co-enriched in the ABC transporters (ko02010) pathway, and both showed an upward trend. *GolS* encodes galactinol synthase, which was also associated with the cytoplasm (GO:0005737), inositol 3-α-galactosyltransferase activity (GO:0047216), carbohydrate storage (GO:0052576), metal ion binding (GO:0046872), the galactose metabolic process (GO:0006012), the cellular response to cold (GO:0070417), and the raffinose family oligosaccharide biosynthetic process (GO:0010325). It catalyzes the critical step of raffinose family oligosaccharide biosynthesis and may play multiple roles in both the response to pathogens and assimilate loading [49]. The caffeoyl-CoA O-methyltransferase (*CCoAOMT*) gene (*LOC110092466*) and sinapyl alcohol were co-enriched in the phenylpropanoid biosynthesis (ko00940) pathway, and both showed a decreasing trend. *CCoAOMT* encodes caffeoyl-CoA O-methyltransferase, which was also associated with metal ion binding (GO:0046872) and O-methyltransferase activity (GO:0008171). In addition, *CCoAOMT* is an important gene that is involved in promoting the clearance of reactive oxygen species and lignin synthesis, thereby conferring drought stress tolerance in *Paeonia ostii* [50]. These results indicate that the metabolism of tyrosine, carbon, and sinapyl alcohol in *D. nobile* reduces to varying degrees. In addition, the ABC transporter pathway was increased to varying degrees in *D. nobile*. Therefore, it can be inferred that there are various regulatory mechanisms in organisms and that metabolites and genes have different regulatory patterns.

Dendrobine is a sesquiterpenoid alkaloid with a typical picrotoxane-type sesquiterpene skeleton structure [16]. The MVA pathway is involved in dendrobine biosynthesis. After MeJA treatment, dendrobine was significantly accumulated (7 d and 14 d). In this study, fourteen terpene synthase genes, twenty-three cytochrome P450 oxidase genes, eight methyltransferase genes, and six aminotransferase genes were found in the downstream pathway that may be involved in dendrobine biosynthesis. The typical plant terpene synthases (TPSs) are a medium-sized gene family with two functions. Class I terpene synthases usually catalyze the cyclization of the lysis of the allylic diphosphate ester bond in acyclic isoprenyl diphosphate precursors [51]. Class II terpene synthases generally catalyze protonation-initiated bicyclization of the diterpene precursor (*E,E,E*)-geranylgeranyl diphosphate (GGPP) to produce *ent*-kaurene [52]. The TPS-e/f and TPS-c subfamilies belong to the class I and class II TPSs, respectively. The typical product of the TPS-e/f subfamily is *ent*-atiseran-16-ol, and the typical products of the TPS-c subfamily are *ent*-copalyl diphosphate, *ent*-kaurene, and *ent*-isopimara-8, 15-diene [51]. In addition, TPSs can be divided into the TPS-a, TPS-b, TPS-d, TPS-g, and TPS-h subfamilies. The major product of the TPS-h subfamily is terpentedienyl-PP, whereas the major product of TPS-a/b/g/d is levopimaradiene [51]. According to the evolutionary analysis, the TPS-d subfamily can be subdivided into TPS-d1, TPS-d2, and TPS-d3. These TPS subfamilies are the basis for the formation of colorful terpenoids.

**Figure 12:**
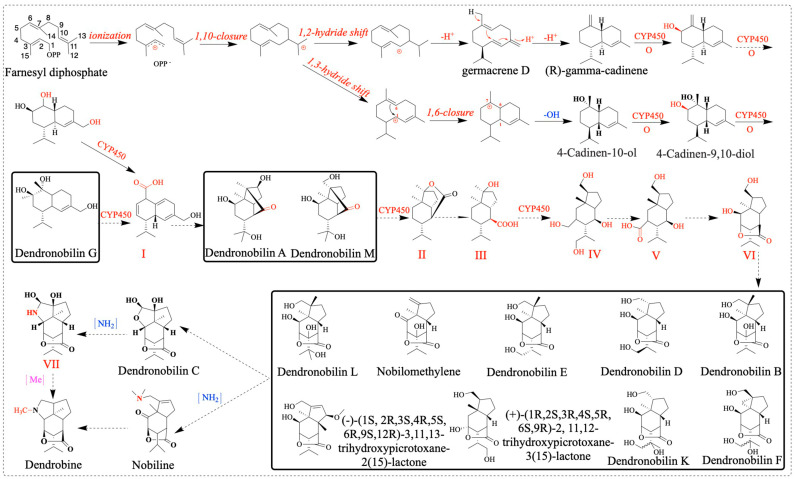
Speculation on the biosynthetic pathway of dendrobine. OPP^−^: diphosphate group, compound of *D. nobile* (inside the bold black box).

Sesquiterpene synthase (*PtTPS5*) has been reported to be a terpene synthase that can catalyze FPP to produce two products, (1S,5S,7R,10R)-guaia-4(15)-en-11-ol and (1S,7R,10R)-guaia-4-en-11-ol as the major products, and hedycaryol as a minor product [53]. In this study, we found a sesquiterpene synthase (*SQS2*) that catalyzes the formation of FPP into different compounds, for example, germacrene D, (*E*)-β-farnesene, α-humulene, β-caryophyllene, 4-hydroxy-germacradiene, nerolidol, germacrene B, germacrene C, and bicyclo-germacrene [31]. *TPS21* also affects dendrobine biosynthesis pathways, so reducing or knocking out this gene may be more beneficial for dendrobine biosynthesis [17]. Combined with the compounds isolated from *D. nobile*, we can further elaborate on the biosynthetic pathway of dendrobine. We speculate the possible cycle–reaction of dendrobine intermediate compounds (dendronobilin G): firstly, under the catalysis of terpene synthases, FPP cyclizes intramolecularly to form dendronobilin G. The intermediate compound of dendrobine is formed through a series of reactions such as molecular rearrangement, oxidation, and cyclization. Cytochrome P450 (*CYP450*) enzymes play significant roles in this pathway. In the dendrobine biosynthesis pathway, we hypothesized that dendronobilin G forms a type I skeleton under the action of CYP450 oxidase. The type I skeleton structures formed by intramolecular rearrangement and cyclization are dendronobilin A and dendronobilin M. These intermediate compounds are further oxidized to form type II skeleton structures. The type II skeleton structures are subsequently hydrolyzed, ring-opened, and oxidized to form type III and IV compound skeleton structures. The picrotoxane-type sesquiterpene skeleton (type V and VI) is formed by intramolecular esterification of the type IV skeleton structure [16]. At least nine picrotoxane-type sesquiterpene compounds have been identified. Picrotoxane-type sesquiterpene compounds (type VI) may be cyclized to form dendronobilin C, which is catalyzed by aminotransferase to form the skeleton VII compound, which is further methylated to form dendrobine [16]. In addition, the picrotoxane-type sesquiterpene compound is directly aminated to nobiline, which is cyclically decarboxylated to dendrobine. The analysis of these methyltransferases has played a role in promoting the biosynthesis of dendrobine. This indicates the direction for us to construct the synthesis pathway of dendrobine and provides a reference for the synthesis of dendrobine (Figure 12).

## 4. Materials and Methods

### 4.1. Plant Materials and Culture Conditions

The seedlings of *Dendrobium nobile* Lindl. were cultivated in the greenhouse of the Institute of Medicinal Plants, Chinese Academy of Medical Sciences and Peking Union Medical College. Plant material was thoroughly sprayed with 50 µM MeJA solution prepared with ddH_2_O containing 0.5% (*v*/*v*) absolute ethanol and then drained and covered with polyethylene bags. The control group was sprayed with the same solution without MeJA; all of the other procedures were the same as those used for the MeJA spray group. After half an hour of treatment, the polyethylene bags were removed and all the plant material was then transferred to the greenhouse. The samples from the experimental and the control groups were collected at 0 h, 0.5 h, 1 h, 2 h, 4 h, 8 h, 16 h, 24 h, 7 d, and 14 d. The samples collected at 0 h, 7 d, and 14 d underwent transcriptome and metabolome analyses and the remaining samples were analyzed for their relative expression levels. These samples were collected, quickly placed in liquid nitrogen, and transferred to an ultra-low-temperature refrigerator for storage. The samples for each stage were prepared with three biological replicates.

### 4.2. Sample Extraction and Wide-Target Metabolomics Analysis

#### 4.2.1. Sample Preparation for Widely Targeted Metabolic Profiling

For the metabolic analysis, eighteen samples were chemically extracted (six groups with three biological replicates). Firstly, biological samples were freeze-dried using a vacuum freeze-drying machine (ScientZ-100F/A, Ningbo, China). Secondly, the lyophilized samples were crushed using a mixed grinder (MM 400, Retsch, Haan, Germany) and zirconia beads at 30 Hz for 1.5 min. Subsequently, 100 mg of freeze-dried powder was dissolved in 1.2 mL of 70% methanol solution, swirled for 30 s every 30 min for a total of six times, and the sample was kept in a refrigerator at 4 °C overnight. Finally, the extract was centrifuged at 12,000 rpm for 10 min and the liquid was filtered (SCAA-104, pore size 0.22 μm; ANPEL, Shanghai, China, http://www.anpel.com.cn/ (accessed on 25 November 2020)), and subjected to an ultrahigh-performance liquid chromatography–tandem mass spectrometry (UPLC–MS/MS) analysis.

#### 4.2.2. Determination of the Dendrobine Content in Samples

The internal standard naphthalene and the dendrobine standard used in the experiment were purchased from the China Food and Drug Control Institute and Sinopharm Chemical Reagent Co., Ltd. (Shanghai, China), respectively. The samples were analyzed in a random order with each injection being 1 μL; each sample was repeated at least three times. For the parameters and methods used for the gas chromatography analysis, we referred to Chinese Pharmacopoeia (2020). Chromatography was performed using an Agilent 6890 GC-FID with an Agilent DB-1 capillary column (0.25 μm × 0.25 mm × 30 m) with nitrogen as the carrier gas. The total ion chromatographic components were determined by flame ionization detection. The relative correction factors for naphthalene and dendrobine were obtained through calculations (f = 0.002836). Finally, the linear regression equation y = 0.13695x + 0.0629 (R^2^ = 0.9997) was obtained, indicating that the concentration of dendrobine was linear, with peak areas ranging from 4.6 to 23.0 mg·L^−1^.

#### 4.2.3. UPLC–MS/MS Conditions

The metabolite data were obtained using the ultrahigh-performance liquid chromatography electrospray tandem triple quadrupole mass spectrometry (UPLC-ESI-MS/MS) system (UPLC, SHIMADZU Nexera X2; MS, Applied Biosystems 4500 Q TRAP, Waltham, MA, USA). The analysis conditions were as follows: the columns used in the UPLC were Agilent SB-C18 columns (1.8 μm, 2.1 mm × 100 mm); the mobile phase solvent A was composed of pure water containing 0.1% formic acid, and solvent B was acetonitrile containing 0.1% formic acid. The samples were detected using a gradient procedure with initial conditions of 95% A and 5% B. Within 9 min, the linear gradient was modified to 5% A and 95% B and a composition of 5% A and 95% B was maintained for 1min. Subsequently, a concentration of 95% A and 5.0% B was adjusted to within 1.10 min and maintained for 2.9 min. The UPLC flow velocity was set as 0.35 mL/min, and the column oven was set to 40 °C; the injection volume was 4 μL (repeated three times for each sample). The effluent was alternatively connected to an ESI-triple quadrupole-linear ion trap (QTRAP)-MS.

#### 4.2.4. Characterization and Quantification of Metabolites

Mass spectrometry collection and the MS/MS analysis were conducted using a mass spectrometer system. Linear ion trap (LIT) and triple quadrupole (QQQ) scans were conducted on the triple quadrupole linear ion trap mass spectrometer (Q TRAP), AB4500 Q TRAP UPLC/MS/MS system. The system is equipped with an ESI Turbo ion spray interface that can be controlled using Analyst 1.6.3 software (AB Sciex, Framingham, MA, USA) to operate in positive and negative ion modes. The operating parameters of the electric spray ion source (ESI) were as follows: the ion source was turbine spray; the ion source temperature was set to 550 °C; the positive and negative ion modes of ion spray voltage (IS) were set at 5500 V and −4500 V, respectively; the curtains gas (CUR), ion source gas I (GSI), and gas II (GSII) were set at 25, 50, and 60 psi, respectively; and the collision-activated dissociation (CAD) was set at high. In the QQQ and LIT modes, mass calibration and instrument tuning were performed with 100 and 10 μM polypropylene glycol solutions, respectively. The collision gas (nitrogen) was set as medium, and QQQ scanning was used in the MRM experiments. The declustering voltage (DP) and collision energy (CE) were further optimized to complete the DP and CE of individual MRM transformations. A specific set of MRM ion pairs was monitored at each period based on the metabolites eluted during each period.

The metabolites were characterized by the existing open metabolomics database, combined with the MetWare database established by Metware Biotechnology Co., Ltd. (Wuhan, China). The metabolites were qualitatively analyzed using MS data with reference to the supplementary mass spectrometry database. These public database sources included KNAPSAcK [54], METLIN [55], MassBank [56], MoToDB [57], and HMDB [58]. Potential biomarker variables were identified by a PCA [59] and OPLS-DA [60]. In addition, differential metabolites could also be further screened by combining the p-values or fold changes from the univariate analysis. A significant difference analysis was performed for potential biomarker variables with a threshold of VIP ≥ 1 and fold changes of ≥2 or ≤0.5. The R software and versions for each analysis were as follows: for the PCA and Pearson’s correlation coefficient analysis, we used R (base package), version 3.5.0; for the heat map analysis, we used R (pheatmap), version 1.0.12; and for the OPLS-DA analysis, we used R (MetaboAnalystR), version 1.0.1.

#### 4.2.5. KEGG Annotation and KEGG Enrichment Analysis of Metabolites

KEGG annotation is the process of comparing and annotating metabolites or genes in the metabolome or transcriptome with pathways, reactions, and genes in the KEGG database. Typically, metabolites to be identified are annotated using the KEGG compound database (http://www.kegg.jp/kegg/compound/ (accessed on 15 December 2020), and the labeled metabolites are then mapped to the KEGG pathway database (http://www.kegg.jp/kegg/pathway.html (accessed on 28 December 2020). Pathways with significantly up- or downregulated metabolites are mapped to the metabolite KEGG network graph and then input into the MSEA (metabolite set enrichment analysis) to determine their significance through the p-value of hypergeometric testing. The metabolome data involved in this study are saved in OMIX, China National Genomics Data Center (https://ngdc.cncb.ac.cn/omix (accessed on 6 December 2021): accession no. OMIX803).

### 4.3. RNA Extraction and Transcriptome Analysis

#### 4.3.1. RNA Extraction and Illumina Sequencing

The total RNA from six samples was extracted and separated by the cetyltrimethylammonium bromide (CTAB) method, with three replicates per sample [61]. The degradation and contamination of the total RNA samples were then assessed by 1% agarose gel electrophoresis. The RNA integrity and concentration were determined using the Agilent 2100 system (Agilent Technologies, Santa Clara, CA, USA) and the NanoDrop 2000/2000C spectrophotometer (Thermo Fisher Scientific, Waltham, MA, USA), respectively. After qualifying the quality detection for the RNA library, different libraries were summarized according to the target data amount and sequenced on the Illumina HiSeq X Ten platform [62]. RNA sequencing was performed for each sample with three biological replicates per sample. RNA sequencing was followed by assembly and analysis at Metware Biotechnology Co., Ltd. (Wuhan, China). Clean data were obtained by filtering the Raw data from the disembarkation and a sequence comparison (HISAT2) was made with *Dendrobium officinale* Kimura et Migo as the reference genome to obtain the mapped data [63,64] before a structural analysis was carried out. The original RNA sequence data were stored in the Genome Sequence Archive (GSA) of the China National Bioinformation Center and the National Genomics Data Center (CNCB-NGDC) under the study entry number CRA005552.

#### 4.3.2. Gene Functional Annotation and Expression Levels Analysis

Unigenes were annotated using Non-Redundant (NR, https://www.merriam-webster.com/dictionary/nonredundant (accessed on 8 January 2021)), the Kyoto Encyclopedia of Genes and Genomes (KEGG) [65], the Protein Family Database (Pfam) [66], The Translation of European Molecular Biology Laboratory (TrEMBL, https://www.embl.org (accessed on 14 January 2021)), Clusters of Orthologous Groups of proteins (KOG, http://clovr.org/docs/clusters-of-orthologous-groups-cogs/ (accessed on 17 January 2021) and https://www.hsls.pitt.edu/obrc/index.php?page=URL1144075392 (accessed on 18 January 2021)), the Protein Sequence Database (SWISS-PROT, http://www.gpmaw.com/html/swiss-prot.html (accessed on 20 January 2021)), and Gene Ontology (GO, http://geneontology.org (accessed on 22 January 2021)). Differentially expressed transcripts for each term were put into the GO mapping database and the transcription of each term was calculated. Through the transcription of this list, a GO function was obtained and the statistics were transcribed. Then, the differentially expressed transcripts (genes) of the GO items were significantly enriched. A similar GO enrichment method was also applied to the KEGG enrichment analysis. KEGG software includes the KEGG API and KGML (v 0.7.2 DTD). To obtain the differential gene set between two biological samples, DESeq2 was used for differential expression analyses between sample groups [67,68,69]. Through a quantitative real-time PCR (qRT-PCR) analysis, 20 candidate genes were selected to study the expression levels. The qRT-PCR primers for 20 candidate gene sequences were designed using Primer Premier 5.0 software, and *DnGAPDH* (F: TAAGGCTGCTATAAAGGAAGAATC; R: GACCTGCTGTCACCC AAGAA) was identified as an internal reference gene. The qRT-PCR analysis was performed using the LightCycler 480 system (Roche, Basel, Switzerland) and the TB Green^®^ Premix Ex Taq^TM^ II (Tli RNaseH Plus) kit (code: RR820A, TAKARA). The 2^−ΔΔCt^ method was used to analyze RT-PCR data and calculate the relative expression levels of the candidate genes [70,71].

#### 4.3.3. Sequence Analysis, Gene Expression in Yeast, and Product Analysis by GC–MS

The target gene (SQS-2) was screened from the transcriptome database, and the full-length primers were designed using Primer Premier 5.0 (the primers are shown in Table S4, Dn-SQS-2-F/R). The primers were sent to GENEWIZ Biotechnology Co., Ltd. (South Plainfield, NJ, USA) for synthesis. The total RNA from the leaves of *D. nobile* was extracted using the CTAB method [61], and the purity and concentration were then measured. Then, the RNA was reverse-transcribed into cDNA using the Thermo Scientifc RevertAid First Strand cDNA Synthesis Kit (Thermo Fisher, Waltham, MA, USA) and the full-length gene was cloned using full-length primers. The target gene was connected to the T vector and sent to GENEWIZ Biotechnology Co., Ltd. for sequencing. The accession no. OR059440 was obtained after submitting the sequence to the NCBI database.

Sequence alignment was performed using BLAST in DNAman and NCBI, and the integrity of the sequence was checked by the ORF finder. Subsequently, the correctly sequenced gene sequence was cloned into the pESC-TRP expression vector (the primers are shown in Table S4, TRP-SQS-2-F/R) to construct the recombinant plasmid. The recombinant plasmid was introduced into *saccharomyces cerevicae* (WAT11) by the lithium acetate method. Then, galactose was added to induce expression, and the extraction liquid was extracted by ethyl acetate after ultrasonic fragmentation. The extraction liquid was dried by a rotary evaporator and then redissolved with 1 mL ethyl acetate. The supernatant was filtered through a 0.22 µm filter and injected into the GC–MS (7890B-5977A; Agilent Technologies, Palo Alto, CA, USA). The test conditions were as follows: manual injection (1 mL), loading on the Agilent J&W HP-5ms column, and elution with N_2_ at 1.0 mL/min. The temperature of the column box was first maintained at 60 °C for 1 min, and then increased to 230 °C at a speed of 10 °C/min, and finally maintained at this temperature for 30 min. The injector and transmission line temperatures were set at 250 °C and 270 °C, respectively. The compounds detected by GC–MS were analyzed using the NIST11 database.

## Figures and Tables

**Table 1:** Classification of the 615 metabolites identified in six groups of *D. nobile*.

Classification	Quantity of Metabolites	Classification	Quantity of Metabolites
Flavonoids	178	Others	24
Phenolic acids	81	Lignans	17
Lipids	77	Quinones	11
Amino acids and derivatives	57	Terpenoids	9
Alkaloids	41	Vitamin	9
Nucleotides and derivatives	37	Stilbene	6
Saccharides and alcohols	33	Tannins	2
Organic acids	32	Xanthone	1

**Table 2:** Summary of the differentially accumulated metabolites (DAMs) and differentially expressed genes (DEGs) among 11 library pairs.

Group	Down ^a^	Up ^a^	Total ^a^	Group	Down ^b^	Up ^b^	Total ^b^
CK_vs_DC7	60	81	141	CK_vs_TC7	233	164	397
CK_vs_DC14	68	49	117	CK_vs_TC14	313	540	853
CK_vs_DF	64	77	141	CK_vs_TF	288	416	704
CK_vs_DF7	68	97	165	CK_vs_TF7	76	116	192
CK_vs_DF14	82	52	134	CK_vs_TF14	624	722	1346
DC7_vs_DC14	82	28	110	TC7_vs_TC14	508	854	1362
DC7_vs_DF7	38	52	90	TC7_vs_TF7	33	96	129
DC14_vs_DF14	49	51	100	TC14_vs_TF14	272	79	351
DF_vs_DF7	27	62	89	TF_vs_TF7	411	366	777
DF_vs_DF14	48	21	69	TF_vs_TF14	212	130	342
DF7_vs_DF14	78	25	103	TF7_vs_TF14	405	484	889

^a^ Metabolites; ^b^ genes.

## Data Availability

The original RNA sequence data were stored in the Genome Sequence Archive (GSA) of the China National Bioinformation Center and the National Genomics Data Center (CNCB-NGDC) with the study entry number CRA005552. The correct sequence of SQS-2 was submitted to NCBI and accession number OR059440 was obtained.

## Supplementary Materials

The following supporting information can be downloaded at: https://www.mdpi.com/article/10.3390/molecules28237892/s1. Figure S1. Content of dendrobine under different concentrations. Figure S2. Sample quality spectrum analysis total ion current diagram. Figure S3. Differential metabolites analysis. Figure S4. Metabolomics profiling of CK_vs_DF7 group. Figure S5. K-means analysis of differentially expressed genes (DEGs). Figure S6. qRT-PCR analysis of 20 differential genes. Figure S7. Reaction catalyzed by (+)- and (−)-germacrene D synthase. Figure S8. Combined transcriptome and metabolome analysis DEGs were significantly correlated with DAMs. Figure S9. Gene cloning, double enzyme digestion of recombinant plasmid agarose gel electrophoresis. Table S1. GO significant enrichment analysis. Table S2. Expression profiles of 238 TFs were corrected with those of structual genes in sesquiterpenoid and triterpenoid biosynthesis. Table S3. Expression profiles of 313 TFs were corrected with those of structual genes in polysaccharides biosynthesis. Table S4. The primers of the twenty selected unigenes. Table S5. Pearson correlation coefficient of DEGs. Table S6. DEGs and DAMs were enriched in the same metabolic pathway and biosynthesis of secondary metabolites pathway.
