# Supplementary material for: The Integration of the Metabolome and Transcriptome for Dendrobium nobile Lindl. in Response to Methyl Jasmonate
Source: Molecules. 2023 Dec 1;28(23):7892. doi: 10.3390/molecules28237892 (PMC10707931; doi:10.3390/molecules28237892)
Supplement: Supplementary file 1 [file molecules-28-07892-s001.zip › Supplement Information.pdf]

Supplement Information

The Integration of the Metabolome and Transcriptome for *Dendrobium nobile* Lindl. in Response to Methyl Jasmonate

Daoyong Gong [1,2], Biao Li [2,*], Bin Wu [2], Deru Fu [3], Zesheng Li [4], Haobo Wei [2,5], Shunxing Guo [2], Gang Ding [2] and Bochu Wang [1,*]

1 College of Bioengineering, Chongqing University, Chongqing 400045, China; 20181901007@cqu.edu.cn
2 Institute of Medicinal Plant Development, Peking Union Medical College, Chinese Academy of Medical Sciences, Beijing 100193, China; bwu@implad.ac.cn (B.W.); 13391950647@163.com (H.W.); sxguo@implad.ac.cn (S.G.); gding@implad.ac.cn (G.D.)
3 Steinhardt School of Culture, Education, and Human Development, New York University, New York, NY 10003, USA; df1978@nyu.edu
4 Dehong Tropical Agriculture Research Institute of Yunnan, Ruili 678600, China; lizesheng120@163.com
5 School of Pharmacy, Chengdu University of Traditional Chinese Medicine, Chengdu 611137, China
* Correspondence: libiao@126.com (B.L.); wangbc@cqu.edu.cn (B.W.)

Supplement Figures/Tables

Figure S1. Content of dendrobine under different concentrations.
Figure S2. Sample quality spectrum analysis total ion current diagram.
Figure S3. Differential metabolites analysis.
Figure S4. Metabolomics profiling of CK_vs_DF7 group.
Figure S5. K-means analysis of differentially expressed genes (DEGs).
Figure S6. qRT-PCR analysis of 20 differential genes.
Figure S7. Reaction catalyzed by (+)- and (-)-germacrene D synthase.
Figure S8. Combined transcriptome and metabolome analysis DEGs were significantly correlated with DAMs.
Figure S9. Gene cloning, double enzyme digestion of recombinant plasmid agarose gel electrophoresis.

Table S1. GO significant enrichment analysis
Table S2. Expression profiles of 238 TFs were corrected with those of structual genes in sesquiterpenoid and triterpenoid biosynthesis.
Table S3. Expression profiles of 313 TFs were corrected with those of structual genes in polysaccharides biosynthesis.
Table S4. The primers of the twenty selected unigenes.

Table S5. Pearson correlation coefficient of DEGs

Table S6. DEGs and DAMs were enriched in the same metabolic pathway and biosynthesis of secondary metabolites pathway.

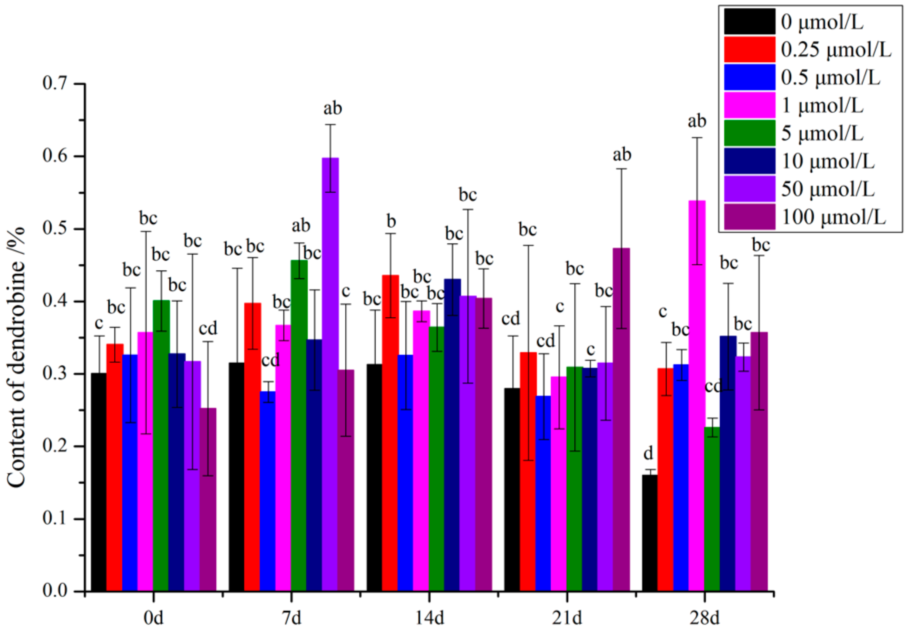

Figure S1. Content of dendrobine under different concentrations.

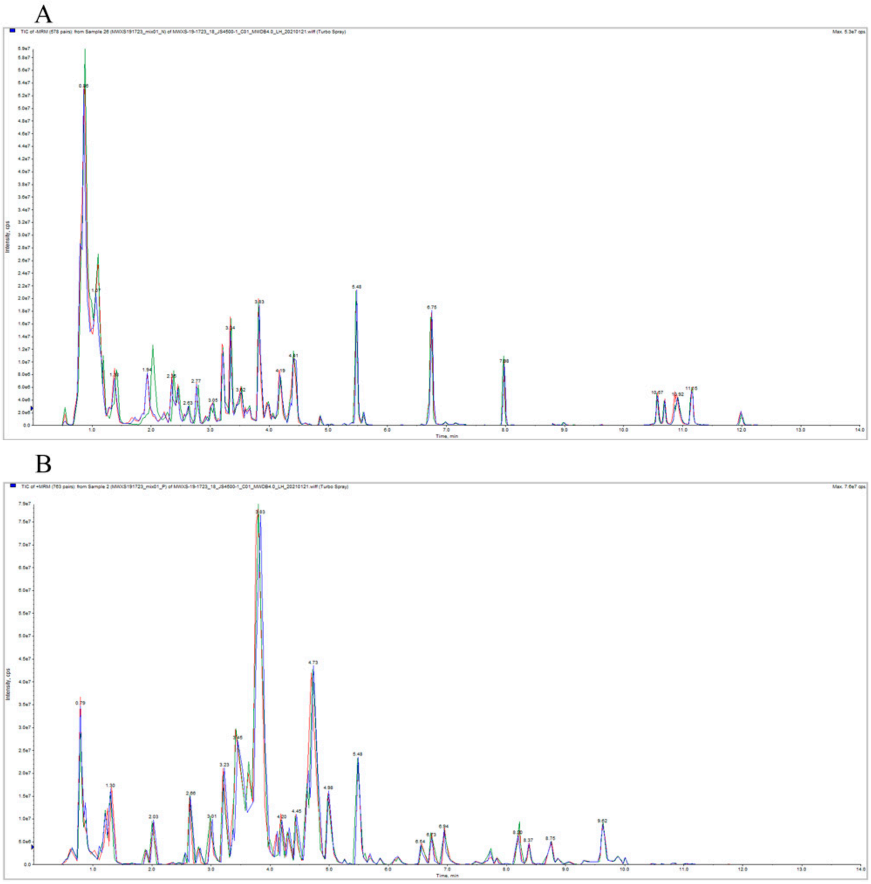

Figure S2. Sample quality spectrum analysis total ion current diagram. Note: N represents negative ion mode (A), P represents positive ion mode (B).

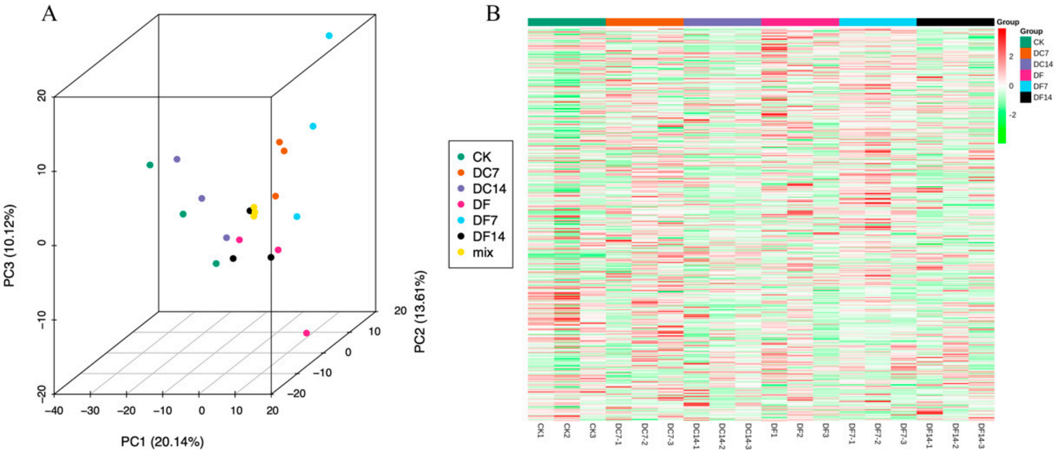

Figure S3. Differential metabolites analysis. (A) PCA 3D plot; (B) Heatmap based on classification analysis. PC1 represents the first principal component, PC2 represents the second principal component, PC3 represents the third principal component, and percentage represents the interpretation rate of this principal component to the data set. Each dot in the diagram represents a sample, and samples from the same group are represented in the same color.

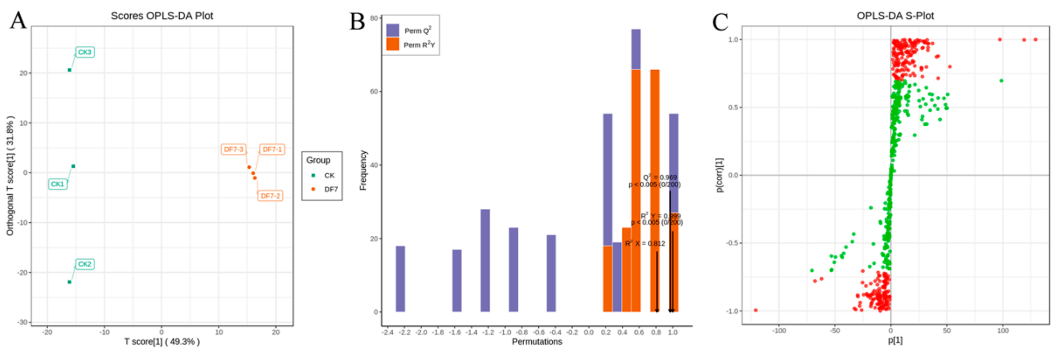

Figure S4. Metabolomics profiling of CK_vs_DF7 group. **(A) Orthogonal Partial Least Squares-Discriminant Analysis (OPLS-DA) score.** PC1 represents the first principal component, PC2 represents the second principal component, PC3 represents the third principal component, and percentage represents the interpretation rate of this principal component to the data set. Each dot in the diagram represents a sample, and samples from the same group are represented in the same color. **(B) OPLS-DA validation model.** The X-axis represents model accuracy, and the Y-axis represents the frequency of model classification effect. Q^2 is an important parameter for evaluating the OPLS-DA model, and the R^2Y and R^2X represent the percentage of OPLS-DA model that can explain Y and X matrix information, respectively; **(C) OPLS-DA S-plot.** X-axis represents the covariance between the principal component and the metabolite, Y-axis represents the correlation coefficient between the principal component and the metabolite. The closer to the upper right and lower left corner of the metabolite, the more significant the difference is. The red dot indicates that the VIP value of the metabolite ≥1, and the green dot indicates that the VIP value of the metabolite <1.

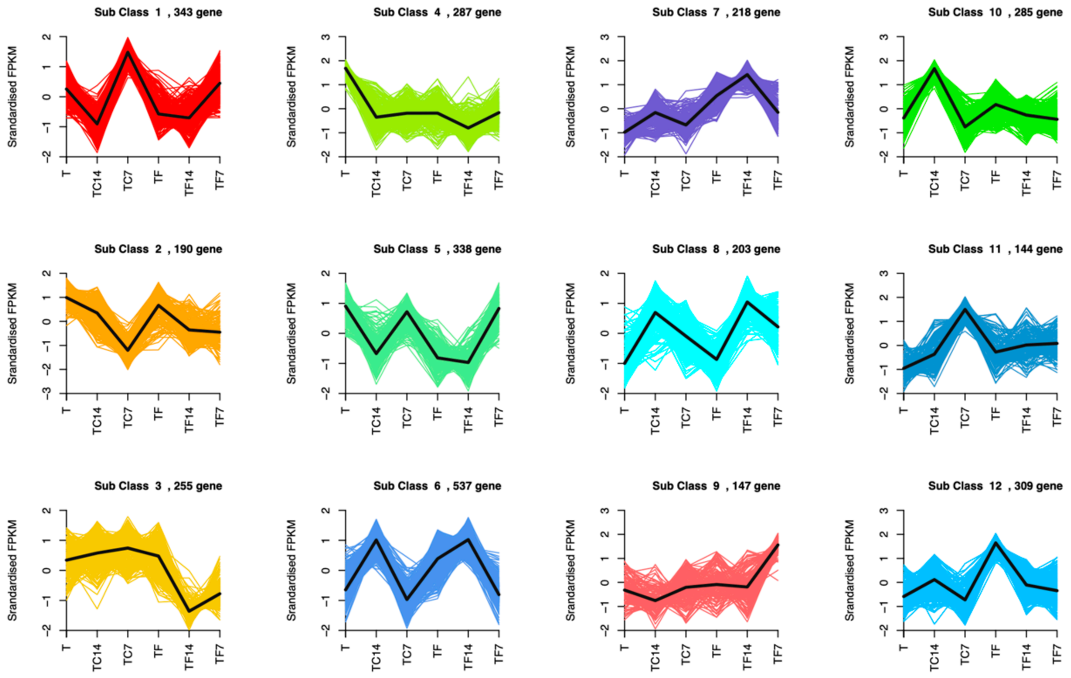

Figure S5. K-means analysis of differentially expressed genes (DEGs). The horizontal coordinate represents the sample name, The ordinate represents centralized and standardized representation.

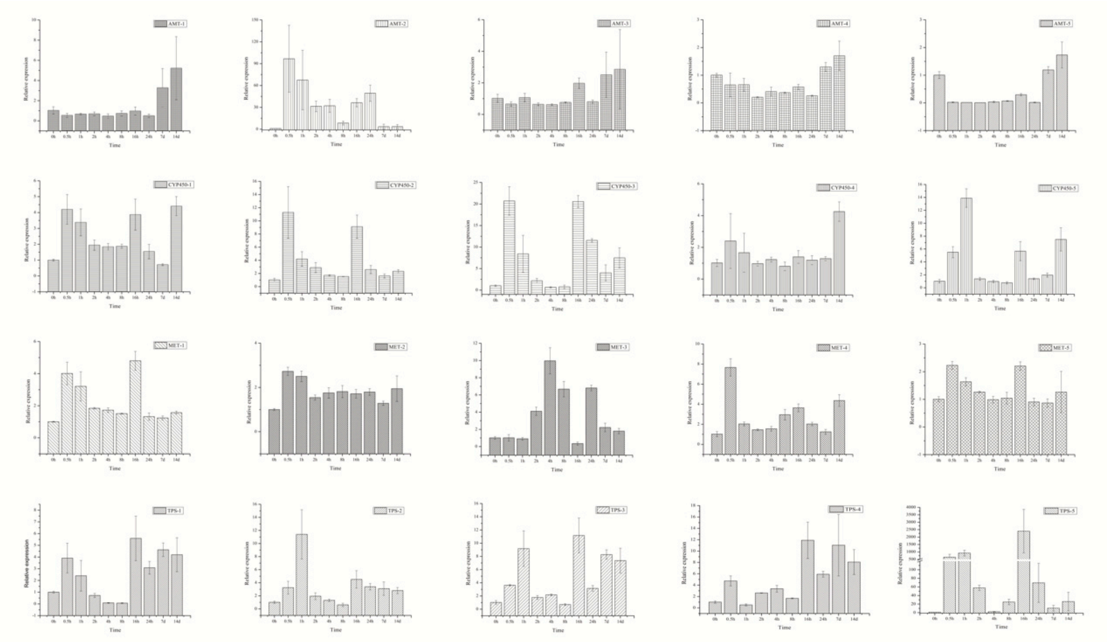

Figure S6. qRT-PCR analysis of 20 differential genes.

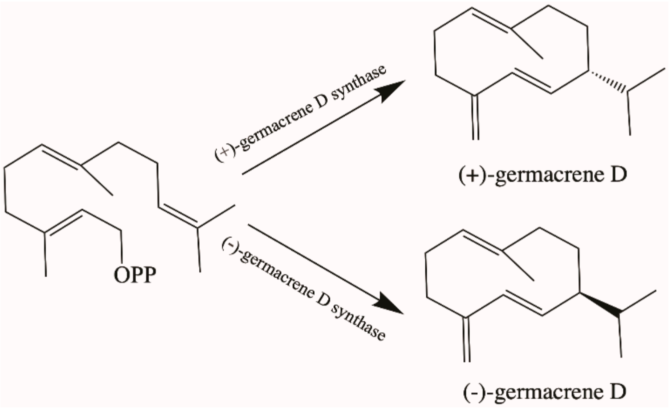

Figure S7. Reaction catalyzed by (+)- and (-)-germacrene D synthase

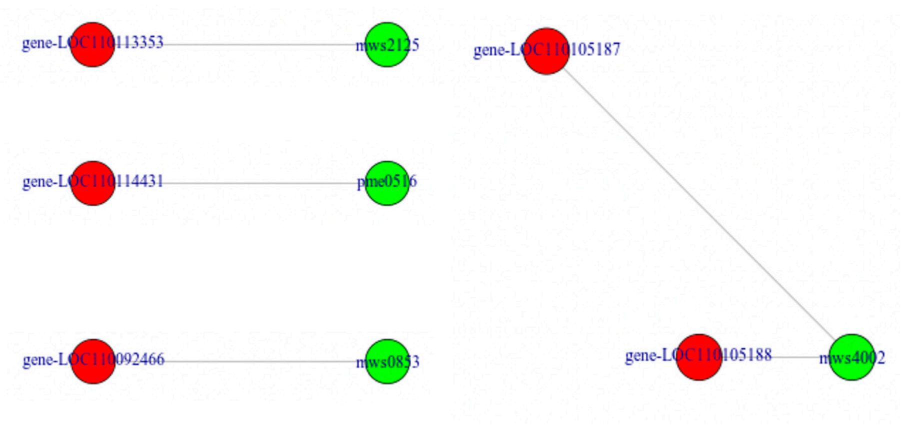

Figure S8. Combined transcriptome and metabolome analysis DEGs were significantly correlated with DAMs.

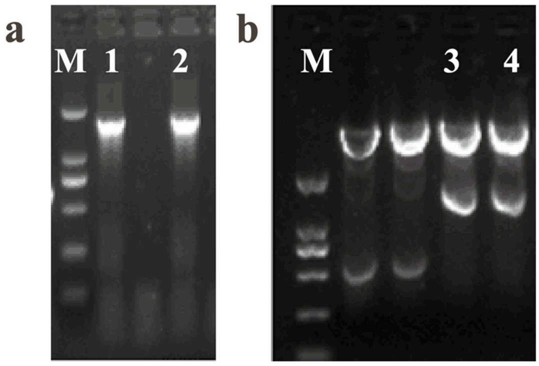

Figure S9. Gene cloning, double enzyme digestion of recombinant plasmid agarose gel electrophoresis.

(M: Marker DL2000; 1,2:SQS-2; 3,4: pESC-TRP::SQS-2 plasmid double enzyme digestion)

Table S1. GO significant enrichment analysis

Ontology	ID	Description	p-value
Molecular function	GO:0004497	monooxygenase activity	5.7027E-07
Molecular function	GO:0016705	oxidoreductase activity, acting on paired donors, with incorporation or reduction of molecular oxygen	2.5835E-06
Molecular function	GO:0016709	oxidoreductase activity, acting on paired donors, with incorporation or reduction of molecular oxygen, NAD(P)H as one donor, and incorporation of one atom of oxygen	2.504E-05
Molecular function	GO:0016413	O-acetyltransferase activity	2.8046E-05
Molecular function	GO:0008374	O-acyltransferase activity	2.9955E-05
Molecular function	GO:1990538	xylan O-acetyltransferase activity	5.8481E-05
Molecular function	GO:0005506	iron ion binding	0.00011032
Molecular function	GO:0016747	transferase activity, transferring acyl groups other than amino-acyl groups	0.00012436
Molecular function	GO:0016746	transferase activity, transferring acyl groups	0.000302
Molecular function	GO:0016407	acetyltransferase activity	0.00073844
Molecular function	GO:0015293	symporter activity	0.00077344
Molecular function	GO:0051119	sugar transmembrane transporter activity	0.0009141
Molecular function	GO:0008509	anion transmembrane transporter activity	0.00099502
Molecular function	GO:0008514	organic anion transmembrane transporter activity	0.00276173
Molecular function	GO:0005355	glucose transmembrane transporter activity	0.003118
Molecular function	GO:0015149	hexose transmembrane transporter activity	0.003118
Molecular function	GO:0008519	ammonium transmembrane transporter activity	0.00315397
Molecular function	GO:0015145	monosaccharide transmembrane transporter activity	0.00379825
Molecular function	GO:0005342	organic acid transmembrane transporter activity	0.00445608
Molecular function	GO:0046943	carboxylic acid transmembrane transporter activity	0.00445608
Molecular function	GO:0015144	carbohydrate transmembrane transporter activity	0.00592266
Molecular function	GO:0010427	abscisic acid binding	0.00883624
Molecular function	GO:0020037	heme binding	0.00946042
Molecular function	GO:0009055	electron transfer activity	0.01034164
Molecular function	GO:0005351	carbohydrate:proton symporter activity	0.01048037
Molecular function	GO:0005402	carbohydrate:cation symporter activity	0.01048037

Ontology	ID	Description	p-value
Molecular function	GO:0019901	protein kinase binding	0.01066508
Molecular function	GO:0052689	carboxylic ester hydrolase activity	0.0116871
Molecular function	GO:0043178	alcohol binding	0.01360024
Molecular function	GO:0019840	isoprenoid binding	0.01515263
Molecular function	GO:0046906	tetrapyrrole binding	0.01773133
Molecular function	GO:0005345	purine nucleobase transmembrane transporter activity	0.01915219
Molecular function	GO:0001085	RNA polymerase II transcription factor binding	0.01964896
Molecular function	GO:0015295	solute:proton symporter activity	0.02318337
Molecular function	GO:0015101	organic cation transmembrane transporter activity	0.02532537
Molecular function	GO:0102811	geraniol 10-hydroxylase activity	0.02532537
Molecular function	GO:0042562	hormone binding	0.02723259
Molecular function	GO:0008028	monocarboxylic acid transmembrane transporter activity	0.02738341
Molecular function	GO:0060089	molecular transducer activity	0.02844737
Molecular function	GO:0004864	protein phosphatase inhibitor activity	0.02894973
Molecular function	GO:0019212	phosphatase inhibitor activity	0.02894973
Molecular function	GO:0019900	kinase binding	0.03233188
Molecular function	GO:0015291	secondary active transmembrane transporter activity	0.03303568
Molecular function	GO:0005544	calcium-dependent phospholipid binding	0.03311882
Molecular function	GO:2001070	starch binding	0.03311882
Molecular function	GO:0015294	solute:cation symporter activity	0.03596035
Molecular function	GO:0005200	structural constituent of cytoskeleton	0.03645485
Molecular function	GO:0015171	amino acid transmembrane transporter activity	0.03797225
Molecular function	GO:0005372	water transmembrane transporter activity	0.04123397
Molecular function	GO:0015205	nucleobase transmembrane transporter activity	0.04123397
Molecular function	GO:0015250	water channel activity	0.04123397
Molecular function	GO:0016411	acylglycerol O-acyltransferase activity	0.04170324
Molecular function	GO:0008324	cation transmembrane transporter activity	0.04584983
Molecular function	GO:0004499	N,N-dimethylaniline monooxygenase activity	0.04832199
Molecular function	GO:0022838	substrate-specific channel activity	0.04977394
Cellular component	GO:0009534	chloroplast thylakoid	0.00489229
Cellular component	GO:0031976	plastid thylakoid	0.00489229
Cellular component	GO:0009705	plant-type vacuole membrane	0.01055567
Cellular component	GO:0009579	thylakoid	0.01075106
Cellular component	GO:0000325	plant-type vacuole	0.01178442
Cellular component	GO:0044436	thylakoid part	0.01224638
Cellular component	GO:0009535	chloroplast thylakoid membrane	0.0140754
Cellular component	GO:0055035	plastid thylakoid membrane	0.0140754
Cellular component	GO:0010494	cytoplasmic stress granule	0.01459273
Cellular component	GO:0042651	thylakoid membrane	0.02727485
Cellular component	GO:0034357	photosynthetic membrane	0.02834208
Cellular component	GO:0005615	extracellular space	0.04233129
Cellular component	GO:0005887	integral component of plasma membrane	0.0433609
Biological process	GO:0048511	rhythmic process	3.5467E-07

Ontology	ID	Description	p-value
Biological process	GO:0007623	circadian rhythm	2.6222E-06
Biological process	GO:0045492	xylan biosynthetic process	4.6993E-06
Biological process	GO:0080167	response to karrikin	8.4672E-06
Biological process	GO:0045491	xylan metabolic process	1.28E-05
Biological process	GO:1900140	regulation of seedling development	1.5181E-05
Biological process	GO:0010029	regulation of seed germination	3.0298E-05
Biological process	GO:0015696	ammonium transport	3.3349E-05
Biological process	GO:0010410	hemicellulose metabolic process	3.3809E-05
Biological process	GO:0015695	organic cation transport	6.286E-05
Biological process	GO:0009639	response to red or farred light	6.7864E-05
Biological process	GO:0070592	cell wall polysaccharide biosynthetic process	7.9312E-05
Biological process	GO:0009411	response to UV	9.3821E-05
Biological process	GO:0044038	cell wall macromolecule biosynthetic process	9.4857E-05
Biological process	GO:0070589	cellular component macromolecule biosynthetic process	9.4857E-05
Biological process	GO:0042546	cell wall biogenesis	0.00014506
Biological process	GO:0010383	cell wall polysaccharide metabolic process	0.00015954
Biological process	GO:0008643	carbohydrate transport	0.00020591
Biological process	GO:0010099	regulation of photomorphogenesis	0.00022933
Biological process	GO:0015995	chlorophyll biosynthetic process	0.00036413
Biological process	GO:0015711	organic anion transport	0.0005419
Biological process	GO:0006820	anion transport	0.00056269
Biological process	GO:0018107	peptidyl-threonine phosphorylation	0.00061238
Biological process	GO:0018210	peptidyl-threonine modification	0.00061238
Biological process	GO:0071478	cellular response to radiation	0.00061988
Biological process	GO:0015994	chlorophyll metabolic process	0.00062494
Biological process	GO:0009637	response to blue light	0.0007323
Biological process	GO:0009739	response to gibberellin	0.00089553
Biological process	GO:0071214	cellular response to abiotic stimulus	0.00106256
Biological process	GO:0104004	cellular response to environmental stimulus	0.00106256
Biological process	GO:0015849	organic acid transport	0.00109557
Biological process	GO:0046942	carboxylic acid transport	0.00109557
Biological process	GO:0006779	porphyrin-containing compound biosynthetic process	0.00115607
Biological process	GO:0071491	cellular response to red light	0.00116628
Biological process	GO:0009845	seed germination	0.00123073
Biological process	GO:0044036	cell wall macromolecule metabolic process	0.00133822
Biological process	GO:0009902	chloroplast relocation	0.0013734
Biological process	GO:0051667	establishment of plastid localization	0.0013734
Biological process	GO:0009640	photomorphogenesis	0.00169524
Biological process	GO:0033014	tetrapyrrole biosynthetic process	0.00169524
Biological process	GO:0019750	chloroplast localization	0.00174677
Biological process	GO:0051644	plastid localization	0.00174677
Biological process	GO:0090351	seedling development	0.00190556
Biological process	GO:0071482	cellular response to light stimulus	0.00211165

Ontology	ID	Description	p-value
Biological process	GO:0006778	porphyrin-containing compound metabolic process	0.00221179
Biological process	GO:0010162	seed dormancy process	0.00242312
Biological process	GO:0022611	dormancy process	0.00242312
Biological process	GO:0033013	tetrapyrrole metabolic process	0.00271261
Biological process	GO:0010114	response to red light	0.00286368
Biological process	GO:2000030	regulation of response to red or far red light	0.00291075
Biological process	GO:0048580	regulation of post-embryonic development	0.002993
Biological process	GO:0071489	cellular response to red or far red light	0.00304619
Biological process	GO:0046148	pigment biosynthetic process	0.00309115
Biological process	GO:0008300	isoprenoid catabolic process	0.00319868
Biological process	GO:0008645	hexose transmembrane transport	0.00335
Biological process	GO:0015749	monosaccharide transmembrane transport	0.00335
Biological process	GO:0046323	glucose import	0.00335
Biological process	GO:1904659	glucose transmembrane transport	0.00335
Biological process	GO:0042440	pigment metabolic process	0.00335318
Biological process	GO:0006721	terpenoid metabolic process	0.00345237
Biological process	GO:0009658	chloroplast organization	0.00412184
Biological process	GO:0006720	isoprenoid metabolic process	0.00564864
Biological process	GO:0009827	plant-type cell wall modification	0.00583986
Biological process	GO:0071483	cellular response to blue light	0.00583986
Biological process	GO:0010187	negative regulation of seed germination	0.00675726
Biological process	GO:0046164	alcohol catabolic process	0.00675726
Biological process	GO:0034754	cellular hormone metabolic process	0.00698536
Biological process	GO:0010380	regulation of chlorophyll biosynthetic process	0.00836199
Biological process	GO:0009834	plant-type secondary cell wall biogenesis	0.00853602
Biological process	GO:0010224	response to UV-B	0.00853602
Biological process	GO:0034637	cellular carbohydrate biosynthetic process	0.00868713
Biological process	GO:0015979	photosynthesis	0.00935005
Biological process	GO:0034219	carbohydrate transmembrane transport	0.00963319
Biological process	GO:0009682	induced systemic resistance	0.01004406
Biological process	GO:1901463	regulation of tetrapyrrole biosynthetic process	0.01004406
Biological process	GO:1901616	organic hydroxy compound catabolic process	0.01170662
Biological process	GO:0033692	cellular polysaccharide biosynthetic process	0.0123113
Biological process	GO:0016114	terpenoid biosynthetic process	0.01405287
Biological process	GO:0090056	regulation of chlorophyll metabolic process	0.01410406
Biological process	GO:0048826	cotyledon morphogenesis	0.01432062
Biological process	GO:0015718	monocarboxylic acid transport	0.01487991
Biological process	GO:0009606	tropism	0.01564549
Biological process	GO:0009644	response to high light intensity	0.01566841
Biological process	GO:0010030	positive regulation of seed germination	0.01582681
Biological process	GO:0010117	photoprotection	0.01582681
Biological process	GO:0010207	photosystem II assembly	0.01582681
Biological process	GO:0009959	negative gravitropism	0.01729916

Ontology	ID	Description	p-value
Biological process	GO:0006865	amino acid transport	0.01763718
Biological process	GO:2000026	regulation of multicellular organismal development	0.01796608
Biological process	GO:0008299	isoprenoid biosynthetic process	0.01878921
Biological process	GO:1901401	regulation of tetrapyrrole metabolic process	0.01918521
Biological process	GO:0055081	anion homeostasis	0.01934521
Biological process	GO:0006863	purine nucleobase transport	0.01999062
Biological process	GO:0009903	chloroplast avoidance movement	0.01999062
Biological process	GO:0043692	monoterpene metabolic process	0.01999062
Biological process	GO:0043693	monoterpene biosynthetic process	0.01999062
Biological process	GO:0009657	plastid organization	0.02187379
Biological process	GO:0044264	cellular polysaccharide metabolic process	0.02187379
Biological process	GO:0010074	maintenance of meristem identity	0.0218963
Biological process	GO:0055088	lipid homeostasis	0.0218963
Biological process	GO:0051193	regulation of cofactor metabolic process	0.02315934
Biological process	GO:0033383	geranyl diphosphate metabolic process	0.02476807
Biological process	GO:0051239	regulation of multicellular organismal process	0.02519805
Biological process	GO:0042752	regulation of circadian rhythm	0.02580233
Biological process	GO:0009751	response to salicylic acid	0.02605175
Biological process	GO:0006535	cysteine biosynthetic process from serine	0.02619779
Biological process	GO:0010222	stem vascular tissue pattern formation	0.02619779
Biological process	GO:0042126	nitrate metabolic process	0.02859476
Biological process	GO:0042128	nitrate assimilation	0.02859476
Biological process	GO:0044262	cellular carbohydrate metabolic process	0.02935018
Biological process	GO:0071669	plant-type cell wall organization or biogenesis	0.02961291
Biological process	GO:0010588	cotyledon vascular tissue pattern formation	0.03017613
Biological process	GO:0019344	cysteine biosynthetic process	0.03017613
Biological process	GO:0009630	gravitropism	0.03092534
Biological process	GO:0009611	response to wounding	0.03167289
Biological process	GO:0010017	red or far-red light signaling pathway	0.03243727
Biological process	GO:0009629	response to gravity	0.03373528
Biological process	GO:0042545	cell wall modification	0.03373528
Biological process	GO:0009862	systemic acquired resistance, salicylic acid mediated signaling pathway	0.03423764
Biological process	GO:0010225	response to UV-C	0.03423764
Biological process	GO:0015804	neutral amino acid transport	0.03423764
Biological process	GO:0015851	nucleobase transport	0.03622598
Biological process	GO:0015698	inorganic anion transport	0.03987303
Biological process	GO:0046677	response to antibiotic	0.04043052
Biological process	GO:0048825	cotyledon development	0.04148536
Biological process	GO:0019827	stem cell population maintenance	0.04215993
Biological process	GO:0098727	maintenance of cell number	0.04215993
Biological process	GO:0010492	maintenance of shoot apical meristem identity	0.04292327
Biological process	GO:0071472	cellular response to salt stress	0.04292327
Biological process	GO:0009268	response to pH	0.04339777

Ontology	ID	Description	p-value
Biological process	GO:0009690	cytokinin metabolic process	0.04369892
Biological process	GO:2000022	regulation of jasmonic acid mediated signaling pathway	0.04961789

Table S2. Expression profiles of 238 TFs were corrected with those of structual genes in sesquiterpenoid and triterpenoid biosynthesis.

Table S3. Expression profiles of 313 TFs were corrected with those of structual genes in polysaccharides biosynthesis.

Table S4. The primers of the twenty selected unigenes.

Primer name	Gene ID	Forward/ Reverse	Sequence
MET-1-F	gene-LOC110093270	Forward	TCACGGCGCTTCTCAGATTC
MET-1-R	gene-LOC110093270	Reverse	CCAGACGGCCTGTAAGATCC
MET-2-F	gene-LOC110098051	Forward	ACGGTTCTTCTTGCGGACTT
MET-2-R	gene-LOC110098051	Reverse	ATACAGCCGACGACAAGGTG
MET-3-F	gene-LOC110098717	Forward	TTCGCGAAATCTACTCCCCG
MET-3-R	gene-LOC110098717	Reverse	TCGCTGCATGACTTTTTGGC
MET-4-F	gene-LOC110108579	Forward	CTCGATGTCTACTCCGACGC
MET-4-R	gene-LOC110108579	Reverse	GCAATCTTCGCCATCCGTTC
MET-5-F	gene-LOC110110516	Forward	CGAGTCCCGATCACCGTTAG
MET-5-R	gene-LOC110110516	Reverse	AGCCAACGGACAGTGAGATG
AMT-1-F	gene-LOC110092540	Forward	AGCTTTCAAGCGACCGTTCT
AMT-1-R	gene-LOC110092540	Reverse	CCTTCTGCGAACCCGTTAGT
AMT-2-F	gene-LOC110095014	Forward	TTCAGACTGTTGACGTGGCA
AMT-2-R	gene-LOC110095014	Reverse	GTCGGAGCACGGAAGGTTTA
AMT-3-F	gene-LOC110105555	Forward	AACCCCAAGAAGCTTGGACC
AMT-3-R	gene-LOC110105555	Reverse	AAGCTCCCACGCCAAGATTT
AMT-4-F	gene-LOC110106907	Forward	TCGCCGCCAAGAAATTCAAC
AMT-4-R	gene-LOC110106907	Reverse	GTTAGGACCTTCCGCCACAT
AMT-5-F	gene-LOC110112458	Forward	TTCGTGCTGACATGGTTCGT
AMT-5-R	gene-LOC110112458	Reverse	GTTTGGCGTGGAAGAGCAAG
TPS-1-F	gene-LOC110092374	Forward	TCGCCTTCGCCATAAGTGTT

TPS-1-R	gene-LOC110092374	Reverse	AAGCCAACCCAAGCTTTTGC
TPS-2-F	gene-LOC110092382	Forward	CTTGGAGCTTCCTTTGCACC
TPS-2-R	gene-LOC110092382	Reverse	AGGCCATTCCCTTGCAAAAC
TPS-3-F	gene-LOC110097122	Forward	AACATTCGCTCGGAGGTCTG
TPS-3-R	gene-LOC110097122	Reverse	AGACCGAGTCGATCAATGGC
TPS-4-F	gene-LOC110100458	Forward	AGCTTCTTCGTCGCTTTGGA
TPS-4-R	gene-LOC110100458	Reverse	AAAGCATGGCCTATGGCACT
TPS-5-F	gene-LOC110109817	Forward	AAAGGTGATGCCCCTCGTTG
TPS-5-R	gene-LOC110109817	Reverse	TCCTGAAGGTACGCATGAGC
CYP450-1-F	gene-LOC110093050	Forward	TATTCGGAACACAGACCGGC
CYP450-1-R	gene-LOC110093050	Reverse	TTATCAGTCGGCTCGCCATC
CYP450-2-F	gene-LOC110097959	Forward	GCCGCTTCCATAATTCTCGC
CYP450-2-R	gene-LOC110097959	Reverse	AGCTCTTCATGGCAACCACA
CYP450-3-F	gene-LOC110104859	Forward	AACCGCCCTTCTCTTAAGGC
CYP450-3-R	gene-LOC110104859	Reverse	TCACTGCGCGGCTTATAACA
CYP450-4-F	gene-LOC110112006	Forward	GACGGATCTCCTTGCTGCTT
CYP450-4-R	gene-LOC110112006	Reverse	AGCGTAGGGAACGAGTGTTG
CYP450-5-F	gene-LOC110116548	Forward	TTCGAAATAGTCGCCGCTGA
CYP450-5-R	gene-LOC110116548	Reverse	ATTGAGCATCCGCTTCACCT
Dn-SQS-2- F	gene-LOC110109817	Forward	GGGGTACCATGGAGGCTCTTCCTGT
Dn-SQS-2- R	gene-LOC110109817	Reverse	GCTCTAGATCAATCGTTCCTATAAATCC
TRP -SQS-2-F	gene-LOC110109817	Forward	GGGGTACCATGGAGGCTCTTCCTGTACCCCTG
TRP -SQS-2-R	gene-LOC110098717	Reverse	CGGGATCCTCAATCGTTCCTATAAATCCAAAT

Table S5. Pearson correlation coefficient of DEGs

Gene ID	Pearson correlation coefficient	p-value
gene-LOC110092540	0.944	0.01
gene-LOC110095014	0.909	0.05
gene-LOC110105555	0.937	0.01
gene-LOC110106907	0.947	0.01
gene-LOC110112458	0.971	0.01
gene-LOC110093050	0.844	0.05

Gene ID	Pearson correlation coefficient	p-value
gene-LOC110097959	0.921	0.01
gene-LOC110104859	0.951	0.01
gene-LOC110112006	0.981	0.01
gene-LOC110116548	0.987	0.01
gene-LOC110093270	0.82	0.05
gene-LOC110098051	0.979	0.01
gene-LOC110098717	0.975	0.01
gene-LOC110108579	0.993	0.01
gene-LOC110110516	0.971	0.01
gene-LOC110092374	0.94	0.01
gene-LOC110092382	0.992	0.01
gene-LOC110097122	0.911	0.05
gene-LOC110100458	0.988	0.01
gene-LOC110109817	0.877	0.05

Table S6. DEGs and DAMs were enriched in the same metabolic pathway and biosynthesis of secondary metabolites pathway.

KEGG pathway	Ko_ID	Gene Count	Meta Count
Metabolic pathways	ko01100	212	33
Biosynthesis of secondary metabolites	ko01110	137	16
